# A PtS-like 4^2^.8^4^ topology arising from the self-assembly of square-planar nodes *via* organotin units: synthesis and crystal structure of the metal–organic framework {[(PhCH_2_)_3_Sn]_2_Ni(CN)_4_}_*n*_

**DOI:** 10.1107/S2056989026002392

**Published:** 2026-03-17

**Authors:** E. Mothi Paul, Helen Stoeckli-Evans

**Affiliations:** a School of Basic Engineering and Sciences, PSN College of Engineering and Technology, Melathediyoor, Tirunelveli 627 152, Tamil Nadu, India; b Institute of Physics, University of Neuchâtel, Rue Emile-Argand 11, 2000 Neuchâtel, Switzerland; University of Aberdeen, United Kingdom

**Keywords:** crystal structure, metal–organic framework, PtS topology, cyano­metallate, organotin(IV), nickel(II).

## Abstract

The self-assembly of K_2_[Ni(CN)_4_] with (PhCH_2_)_3_SnCl in a 1:2 molar ratio affords the title three-dimensional coordination polymer, poly[hexa­benzyl­tetra-μ-cyanato-nickel(II)ditin(IV)], [NiSn_2_(C_7_H_7_)_6_(CN)_4_]_*n*_ or {[(PhCH_2_)_3_Sn]_2_Ni(CN)_4_}_*n*_. The asymmetric unit is composed of four Ni(CN)_4_ units and eight (PhCH_2_)_3_Sn units. This neutral guest-free metal–organic framework (MOF) has a connectivity defined by the circuit symbol 4^2^.8^4^. The same circuit symbol was originally observed for PtS. The nickel(II) atom in each of the four Ni(CN)_4_ units has a square-planar geometry, while the penta-coordinated Sn atoms have distorted trigonal–pyramidal geometries. The benzyl groups are involved in a number of C—H⋯π inter­actions, which help to consolidate the framework. There are also two pairs of ring–metal inter­actions present involving two nickel atoms.

## Abstract 2

The self-assembly of K_2_[Ni(CN)_4_] with (PhCH_2_)_3_SnCl in a 1:2 molar ratio affords the title three-dimensional coordination polymer. This neutral guest-free metal–organic framework has a three-dimensional connectivity defined by the circuit symbol 4^2^.8^4^.

## 1. Chemical context

The quest for porous mol­ecular materials, exhibiting good stability, high void volumes, and well-defined tailorable cavities, has been the driving force in the synthesis of extended coordination networks for a number of years (Li *et al.*, 1999 ▸; Eddaoudi *et al.*, 2002 ▸; Yaghi *et al.*, 2003 ▸; Kitagawa *et al.*, 2004 ▸; Snurr *et al.*, 2004 ▸; Ferey, 2008 ▸; Bradshaw *et al.*, 2005 ▸). More recently, applications of transition-metal MOFs have been the subject of a study by Wright *et al.* (2025 ▸), and the use of MOFs as anti-corrosive materials has been reviewed by Ansari *et al.* (2025 ▸).

Apart from the formation of various unprecedented network types, many three-dimensional (3D) frameworks or MOFs are based on simple inorganic structures. For example, a diamond-like framework observed for {[N(CH_3_)_4_][Cu^I^Zn^II^(CN)_4_]}_*n*_ (Hoskins & Robson, 1990 ▸), which is one of the first framework coordination polymers to be reported. The PtS-like topology was observed by Abrahams *et al.* (1994 ▸) for a framework structure obtained using porphyrin building blocks. Quartz-like nets were observed in the structure of [ZnAu_2_(CN)_4_]_*n*_ (Hoskins *et al.*, 1995 ▸). In general, the common topologies generated by square-planar nodes vary from simple two-dimensional 4^4^ (square grid) networks (Eckhardt *et al.*, 2000 ▸) to three-dimensional networks such as NbO (6^4^.8^2^; Wells, 1984 ▸) and CdSO_4_ (6^5^.8; Bregeaul & Herpin, 1970 ▸). A twofold inter­penetrating three-dimensional 4^2^.8^4^ network for [Cu(4,4′-bi­pyridine)(NO_3_)_2_]_*n*_, has been described by Lu *et al.* (2005 ▸). Using a ditopic bis­(3,2′:6′,3′′-terpyridine) ligand, Klein *et al.* (2015 ▸) have synthesized a three-dimensional 4^2^.8^4^ network involving octa­hedrally coordinated cobalt(II) square-planar nodes.

Coordination polymers formed with metal cyanide anions and organotin(IV) cations are of considerable inter­est due to their potential applications as porous materials (Fischer *et al.*, 2003 ▸; Niu *et al.*, 1998 ▸). Several two-dimensional (2D) or three-dimensional structures having the general formula [(*R*_3_Sn)*_n_M*(CN)_2*n*_] (*n* = 1–4) are known (Xu *et al.*, 2006 ▸). They exhibit various kinds of topologies arising from the way the polymeric *M*–CN–Sn–NC–*M* chains inter­sect each other. In the compounds formed by square-planar Ni(CN)_4_^2–^ anions and *R*_3_Sn^+^ cations in a 1:2 ratio, two possible structure types are generally anti­cipated and observed. The first is a 2D (4^4^) (square grid) layer structure in which all the Ni(CN)_4_ planes are parallel, as observed in [(Me_3_Sn)_2_Ni(CN)_4_]_*n*_ (Eckhardt *et al.*, 2000 ▸). The second is a 3D **nbo** (6^4^.8^2^) type structure involving alternating planar and perpendicular Ni(CN)_4_ planes. The network in [(Ph_3_Sn)_2_Ni(CN)_4_·Ph_3_SnOH·0.8(MeCN)·0.2(H_2_O)]_*n*_ (Niu *et al.*, 1998 ▸) exhibits such a topology.

Herein, we report on the synthesis and crystal structure of the title compound, {[(PhCH_2_)_3_Sn]_2_Ni(CN)_4_}_*n*_ (**I**), which to the best of our knowledge is the first three-dimensional cyano­metallate coordination polymer that possesses a **pts** (4^2^.8^4^) topology (*CrystalNets*; Zoubritzky & Coudert, 2022 ▸), based solely upon square-planar nodes.
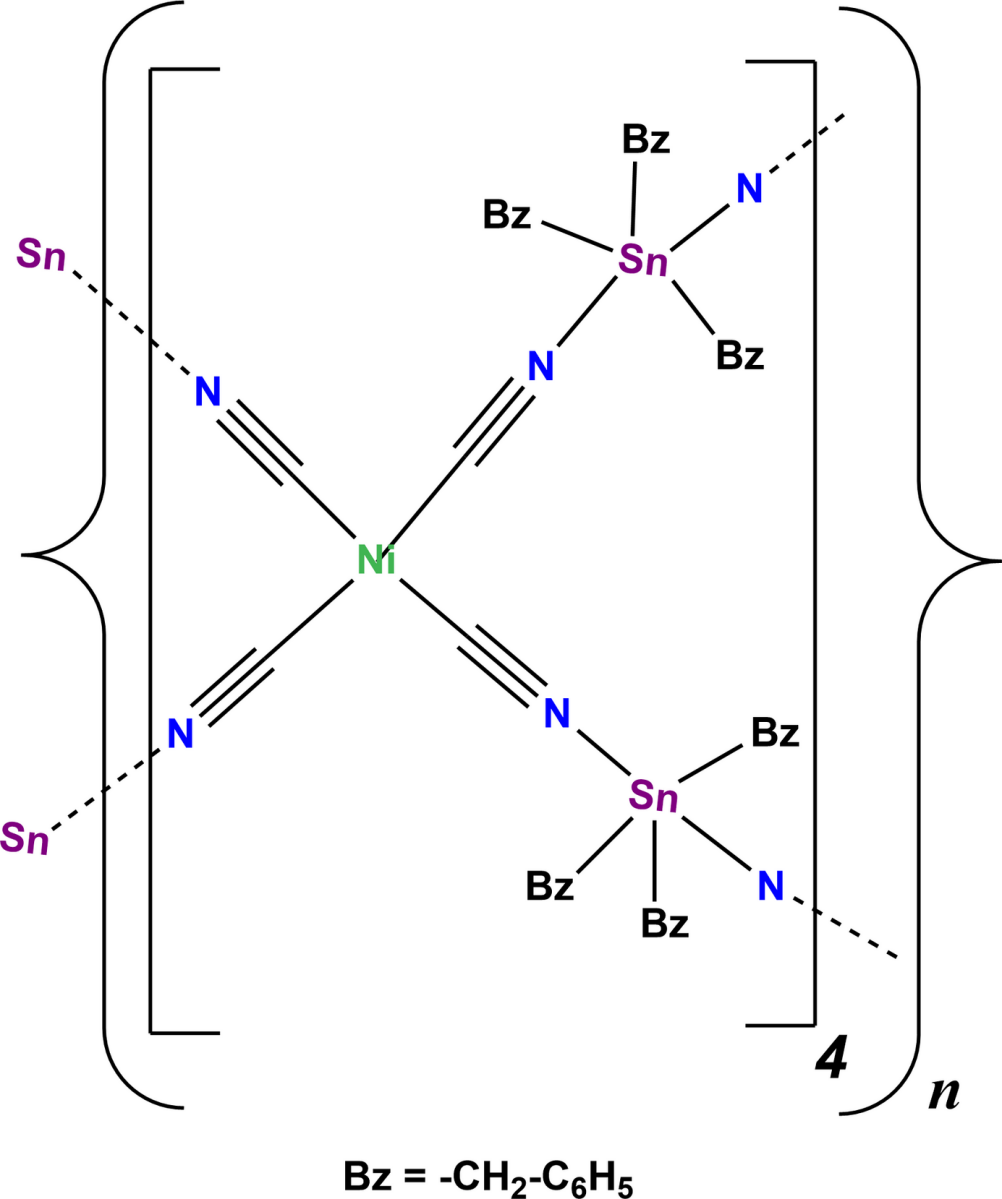


## 2. Structural commentary

**Figure 1:**
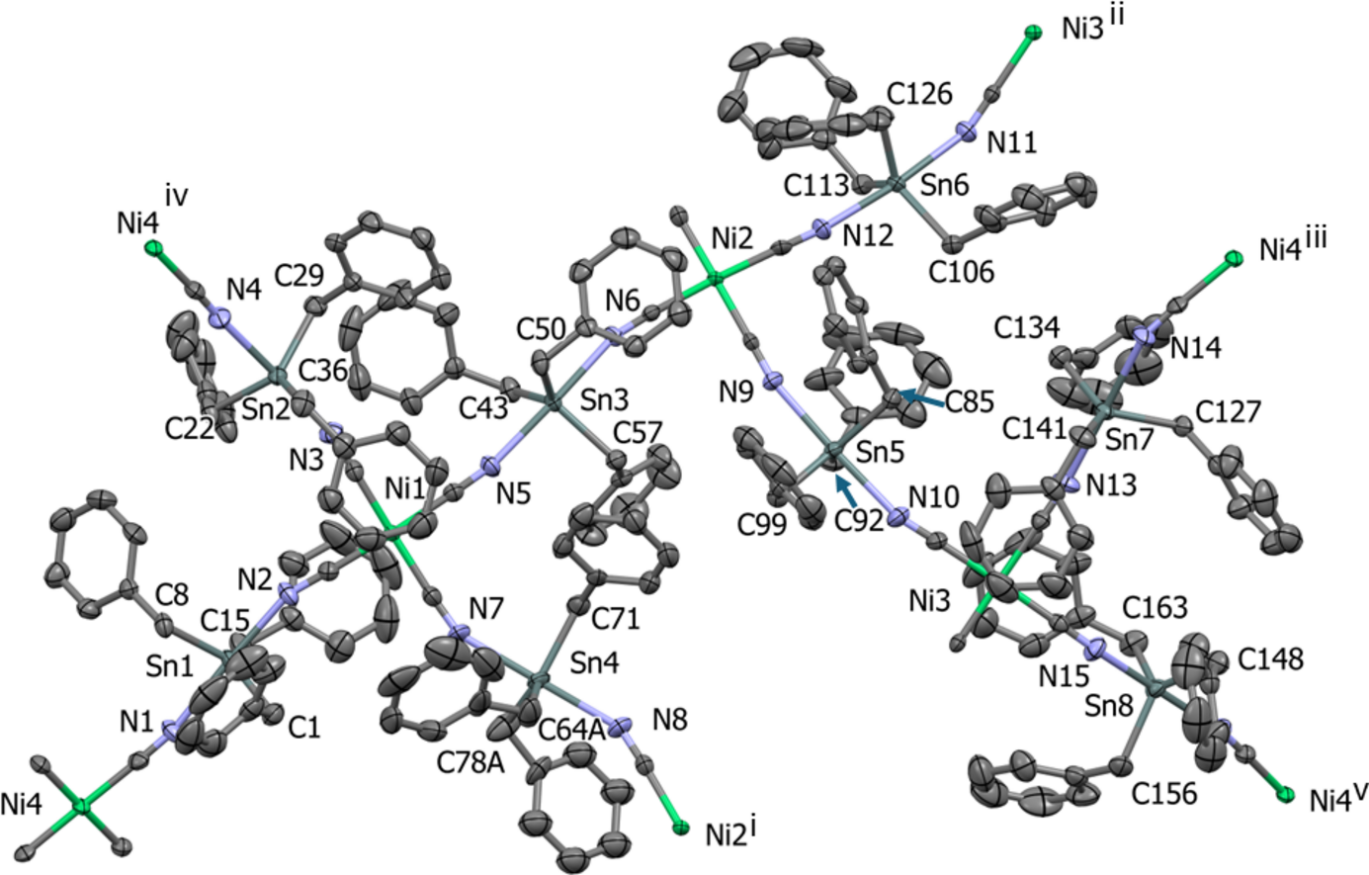
The mol­ecular structure of the asymmetric unit of (**I**). The displacement ellipsoids are drawn at the 50% probability level. For clarity, only the metal, N and methyl­ene C atoms have been labelled, and the minor disorder components of the positionally disordered atoms have been omitted in this and subsequent figures. [Symmetry codes: (i) 1 + *x*, *y*, *z*; (ii) −1 + *x*, *y*, *z*; (iii) −1 + *x*, *y*, 1 + *z*; (iv) 1 − *x*, −*y*, 1 − *z*; (v) 2 − *x*, 1 − *y*, −*z*.]

Compound (**I**), was obtained in single-crystal form by slow inter­diffusion of solutions of K_2_[Ni(CN)_4_] in water and (PhCH_2_)_3_SnCl in aceto­nitrile and crystallizes in the triclinic space group *P*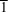. The asymmetric unit is composed of four Ni(CN)_4_ units and eight (PhCH_2_)_3_Sn units (Fig. 1 ▸). Selected geometrical parameters for compound (**I**) are given in Table 1 ▸ and a more exhaustive list is given in Table S1 of the supporting information.

**Figure 2:**
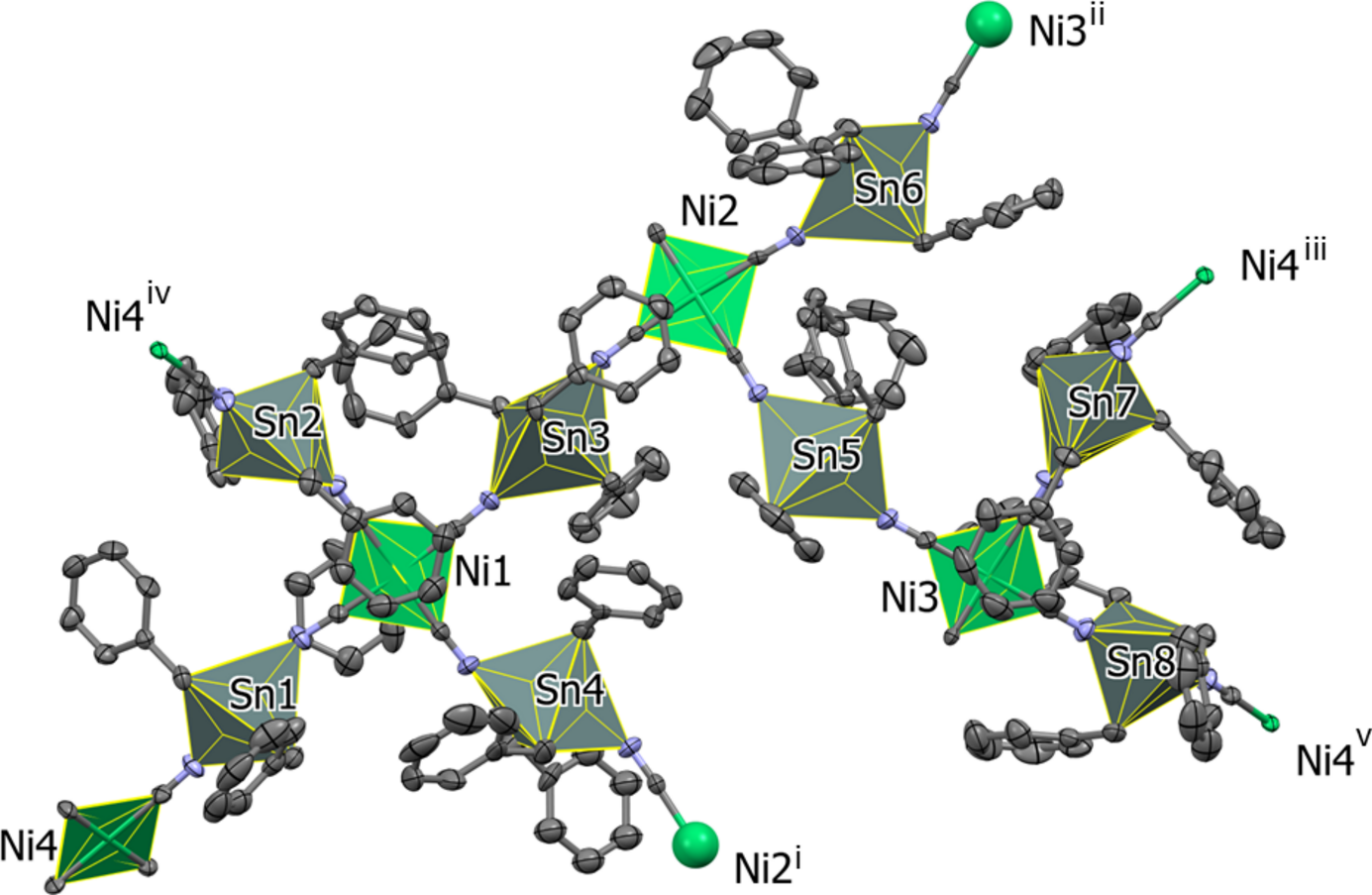
A view of the mol­ecular structure of the asymmetric unit of compound (**I**), showing the Ni and Sn polyhedra. [Symmetry codes: (i) 1 + *x*, *y*, *z*; (ii) −1 + *x*, *y*, *z*; (iii) −1 + *x*, *y*, 1 + *z*; (iv) 1 − *x*, −*y*, 1 − *z*; (v) 2 − *x*, 1 − *y*, −*z*.]

The nickel(II) atom in each of the four Ni(CN)_4_ units has a square-planar geometry (Fig. 2 ▸); two almost perfect and two slightly distorted. The τ_4_ structural indices describing the geometry of atoms Ni1 to Ni4 are 0.10, 0.03, 0.09 and 0.02, respectively (τ_4_ = 1 for a perfect tetra­hedral geometry, = 0 for a perfect square-planar geometry and 0.85 for perfect trigonal–pyramidal geometry; Yang *et al.*, 2007 ▸).

The Sn atoms are penta­coordinated due to the coordination of the nitro­gen atoms of the bridging cyano groups and exhibit distorted trigonal–pyramidal geometries (Fig. 2 ▸); one of the benzyl groups [atoms C78/(C79–C84)] coordinated to atom Sn4 shows positional disorder; see §5, *Refinement.* The τ_5_ structural indices describing the geometry of atoms Sn1 to Sn8 are 0.83, 0.95, 0.83, 0.83, 0.90, 0.88, 0.86 and 0.95, respectively (τ_5_ = 1 for perfect trigonal–pyramidal geometry and = 0 for perfect square-pyramidal geometry; Addison *et al.*, 1984 ▸).

**Figure 3:**
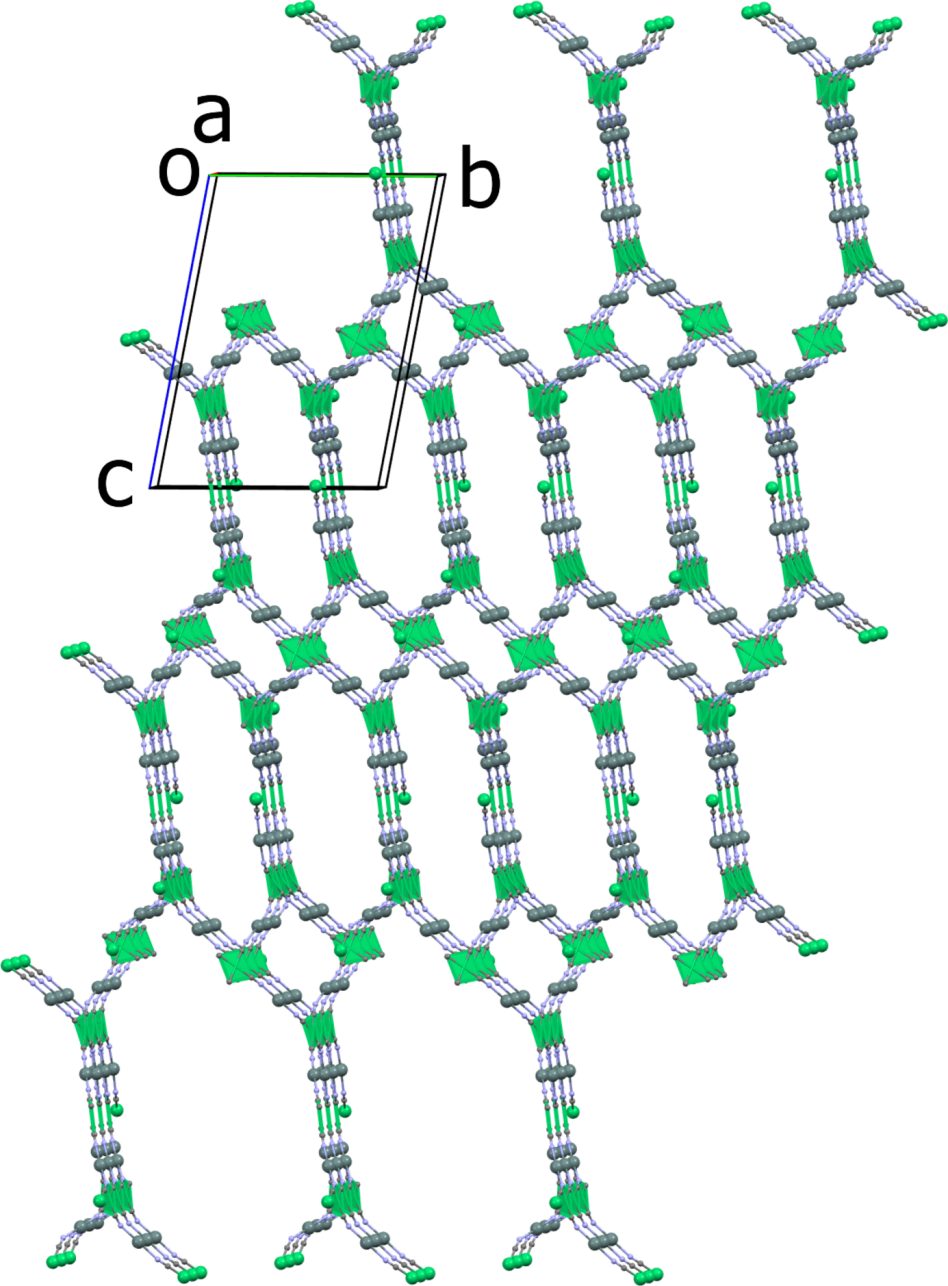
Presentation of the three-dimensional framework of (**I**) viewed along the *a*-axis direction. For clarity, the benzyl groups have been omitted.

The Sn—N distances vary from 2.244 (4) Å for the Sn8—N15 bond to 2.394 (4) Å for the Sn7—N14 bond (Table 1 ▸), compared to the average distance of *ca* 2.33 Å observed in [(Me_3_Sn)_2_Ni(CN)_4_]_*n*_ (Eckhardt *et al.*, 2000 ▸). When one examines the connectivity of the Ni centres, the planes of adjacent square-planar nodes are neither parallel nor perpendicular to each other but show dihedral angles ranging from 19.73 (1) to 34.98 (2)°. This twist of adjacent Ni(CN)_4_ planes results in the formation of the 3D network (see Figs. 3 ▸ and 4 ▸). The circuit symbol for this network (4^2^.8^4^) is similar to that for PtS. However, the PtS network contains both square planar and tetra­hedral nodes in a 1:1 ratio (Wells, 1977 ▸). Another example showing square planar nodes arranging to give a (4^2^.8^4^) network is the polymer [Zn(nicotinate)_2_]n (Rather *et al.*, 2002 ▸). However, this compound has a 3D **lvt** network (*CrystalNets*; Zoubritzky & Coudert, 2022 ▸), hence the projection down [100] is not superimposable upon that of PtS.

## 3. Supra­molecular features

For compound (**I**), the projection down [100] resembles that of PtS (Wells, 1977 ▸), although in (**I**) the hexa­gonal and rectangular channels running along the *a*-axis direction have different dimensions (Figs. 3 ▸ and 4 ▸). The rectangular channels in (**I**) have widths of *ca* 11.02 and 9.73 Å, whereas the hexa­gonal channels show widths of *ca* 11.70 and 9.89 Å, measured across the projection of opposing Sn atoms. The nodes are eclipsed along the *a*-axis direction with a vertical distance between the two Ni atoms of *ca* 14.12 Å. In agreement with the range of twist angles observed in the adjacent Ni planes the C—N—Sn angles exhibit both significantly bent [C208—N8—Sn4 = 152.1 (4)°] and near linear [C204—N4—Sn2 = 173.5 (4)°] arrangements. This is in contrast to the situation in [(Me_3_Sn)_2_Ni(CN)_4_]_*n*_ [178.94 (19)°; Eckhardt *et al.*, 2000 ▸] and [(Ph_3_Sn)_2_Ni(CN)_4_·Ph_3_SnOH·0.8(MeCN)·0.2(H_2_O)]_*n*_ (*ca* 169.6°; Niu *et al.*, 1999 ▸), which show only near linear arrangements.

That no solvent mol­ecules are incorporated into the structure of (**I**) indicates that the benzyl groups bonded to Sn occupy most of the space in the channels within the structure. This is significant when contrasted to the host–guest network [(Ph_3_Sn)_2_Ni(CN)_4_·Ph_3_SnOH·0.8(MeCN)·0.2(H_2_O)] (Niu *et al.*, 1999 ▸), where the presence of a hydrogen-bonded (Ph_3_SnOH)_3_ trimer is shown to template the formation of the 3D network. In compound (**I**), the sole structure directors are most likely the benzyl groups that prevent the formation of an inter­penetrating network.

**Figure 5:**
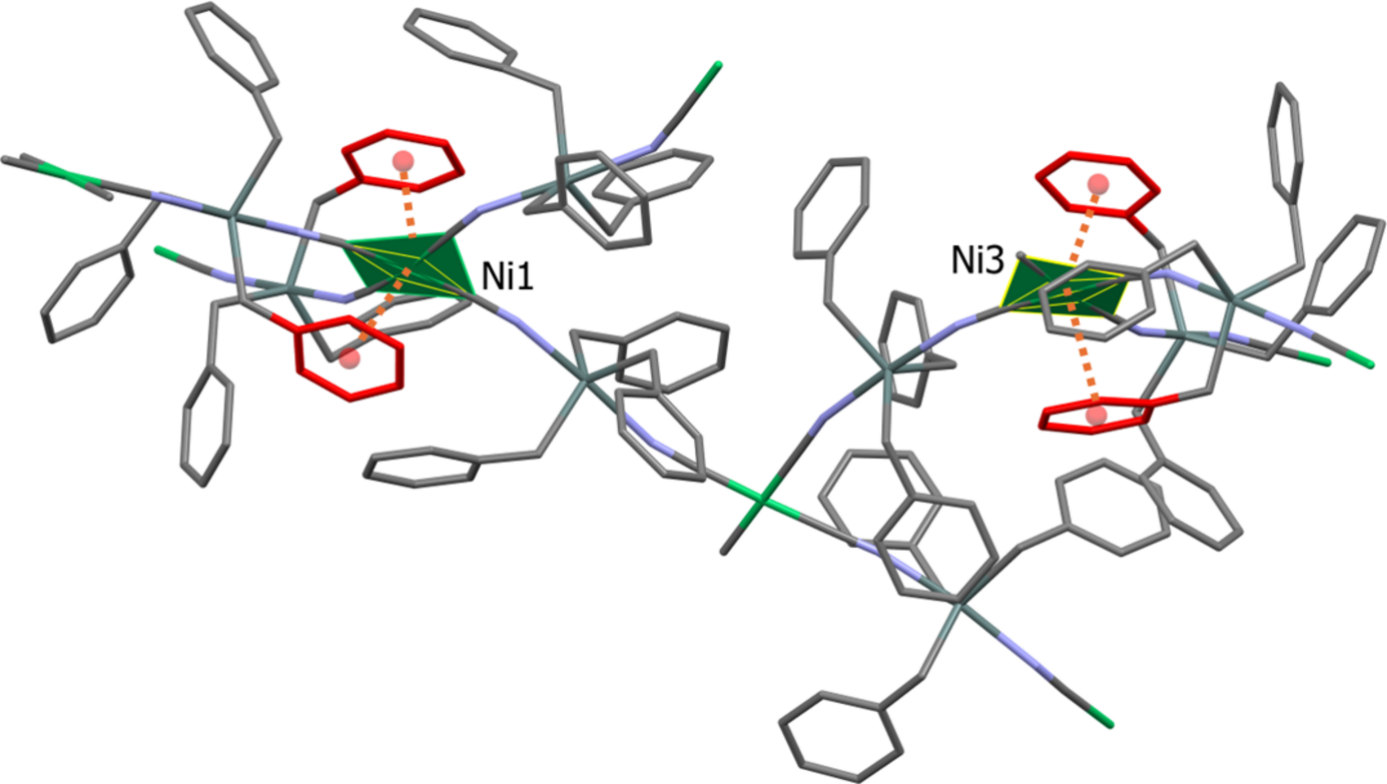
A view of the ring–metal inter­actions involving nickel atoms Ni1 and Ni3.

An analysis of the structure using *PLATON* (Spek, 2020 ▸) indicated that the benzyl groups are involved in a number of C—H⋯π inter­actions (see Table 2 ▸), which help to consolidate the framework. There are also two pairs of ring–metal inter­actions present involving nickel atoms Ni1 and Ni3. This situation is illustrated in Fig. 5 ▸. The first pair involves Ni1⋯*Cg*1 (*Cg*1 is the centroid of the C16–C21 ring) and Ni1⋯*Cg*5 (*Cg*5 is the centroid of the C30–C35 ring); the *Cg*⋯Ni distances being *ca* 3.938 and 3.749 Å, respectively, with the rings being inclined to each other by 14.9 (4)°. The second pair involves Ni3⋯*Cg*21 (*Cg*21 is the centroid of the C142–C147 ring) and Ni3⋯*Cg*24 (*Cg*24 is the centroid of the C164–C169 ring); the *Cg*⋯Ni distances being *ca* 3.729 and 3.663 Å, respectively, here the rings are inclined to each other by 20.1 (3)°. A search of the Cambridge Structural Database (CSD; version 6.01, update November 2025; Groom *et al.*, 2016 ▸) for Ni(CN)_4_ units in a similar environment revealed the presence of two structures (CSD refcodes CADREF: Kuang *et al.*, 2002 ▸; GEKYOL: Cordiner *et al.*, 2006 ▸) where the Ni⋯centroid distances are, however, much shorter at *ca* 3.57 and 3.50 Å, respectively, with inversion-related aromatic rings being parallel to each other in both cases.

In conclusion, we have synthesized and structurally characterized a novel 3D coordination polymer, {[(PhCH_2_)_3_Sn]_2_Ni(CN)_4_}_*n*_, (**I**). This provides the first example of a cyano­metallate network exhibiting a **pts** (4^2^.8^4^) topology (*CrystalNets*; Zoubritzky & Coudert, 2022 ▸), solely based upon square-planar nodes. The structure of this metal–organic framework (MOF) is also unique in the sense that only benzyl groups and not solvent or guest mol­ecules direct the formation of a 3D framework, instead of the expected 2D network. It is possible that different substituents in the benzyl groups could offer still more variety in the resulting networks, which could lead to the formation of porous open frameworks.

## 4. Synthesis and crystallization

K_2_[Ni(CN)_4_] (0.052 g, 0.216 mmol) was dissolved in distilled water (10 ml) and placed in a 3 -ml screw-capped tube. A mixture of water/aceto­nitrile (1/1, 5 ml) was applied as a buffer layer, and a solution of (PhCH_2_)_3_SnCl (0.277 g, 0.648 mmol) in aceto­nitrile (10 ml) was layered on top. Colourless crystals of the title compound were isolated from the inter­face after about 3 weeks of inter­diffusion. Yield: 0.11 g (53% based on K_2_[Ni(CN)_4_]). Elemental analysis (%) calculated for (**1)**: C, 53.84; H, 4.47; N, 5.92, found: C, 53.62; H, 4.40; N, 5.89. IR (KBr, cm^−1^): 3434 (*m*), 3079 (*m*), 2142 (*s*, *ν*_CN_), 1491 (*m*), 1451 (*m*), 1053 (*m*), 756 (*s*), 695 (*s*). The IR spectrum of (**1**) shows the characteristic C=N stretching band at 2142 cm^−1^. Such an upward (blue) shift of this band from that of K_2_[Ni(CN)_4_] at 2127 cm^−1^ is a common feature in organotin–metal cyanide complexes (Niu *et al.*, 1998 ▸; Brimah *et al.*, 1994 ▸; Bonardi *et al.*, 1991 ▸).

## 5. Refinement

Crystal data, data collection and structure refinement details are summarized in Table 3 ▸. The C-bound H atoms were included in calculated positions and refined as riding atoms; C—H = 0.95–0.99 Å, *U*_iso_(H) = 1.2*U*_eq_(C). One of the methyl­ene groups (C64) and a benzyl group [atoms C78/(C79–C84)], undergo positional disorder; see Fig. S1 of the supporting information. The occupancies of both groups and the attached H atoms were freely refined; giving occupancies of 0.69 (4):0.31 (4) for C64*A*:C64*B*, and 0.51 (1):0.49 (1) for C78*A*–C84*A*:C78*B*–C84*B*. Benzene rings C65–C70, C79*A*–C84*A* and C79*B*–C84*B* were refined as regular hexa­gons *i.e.* rigid groups with a C—C separation of 1.39 Å.

## Supplementary Material

Crystal structure: contains datablock(s) I, global. DOI: 10.1107/S2056989026002392/hb8199sup1.cif

Structure factors: contains datablock(s) I. DOI: 10.1107/S2056989026002392/hb8199Isup2.hkl

Table S1. Selected bond lengths and bond angles for compound (I) . Figure S1. A view of the molecular structure of the asymmetric unit of compound (I). DOI: 10.1107/S2056989026002392/hb8199sup3.pdf

CCDC reference: 704360

Additional supporting information:  crystallographic information; 3D view; checkCIF report

## Figures and Tables

**Figure 4:**
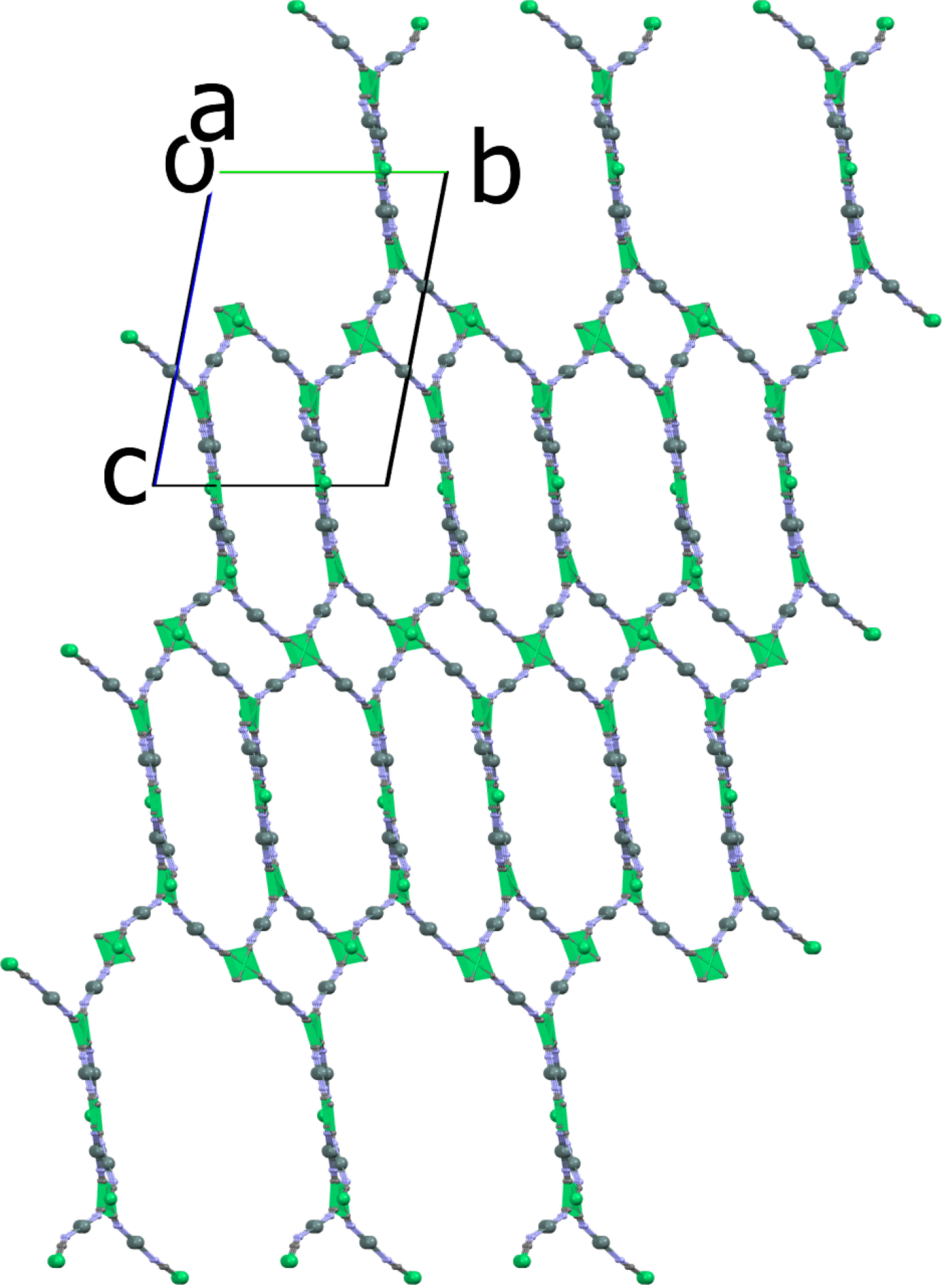
Illustration of the (4^2^.8^4^) topology. The highlighted [Ni(CN)_4_]^2−^ square planes (green) indicate the twists leading to the three-dimensional framework. For clarity, the benzyl groups have been omitted.

**Table 1:** Selected geometric parameters (Å, °)

Sn1—N1	2.373 (4)	Sn5—N9	2.303 (4)
Sn1—N2	2.274 (4)	Sn5—N10	2.335 (4)
Sn2—N3	2.273 (4)	Sn6—N11	2.340 (4)
Sn2—N4	2.359 (4)	Sn6—N12	2.296 (4)
Sn3—N5	2.321 (4)	Sn7—N13	2.260 (3)
Sn3—N6	2.330 (3)	Sn7—N14	2.394 (4)
Sn4—N7	2.311 (4)	Sn8—N15	2.244 (4)
Sn4—N8	2.342 (4)	Sn8—N16	2.387 (4)
			
C202—Ni1—C205	172.7 (2)	C141—Sn7—C134	127.0 (2)
C203—Ni1—C207	172.7 (2)	N13—Sn7—N14	178.39 (16)
C209—Ni2—C208^i^	178.9 (2)	C148—Sn8—C163	122.4 (2)
C212—Ni2—C206	177.2 (2)	N15—Sn8—N16	179.58 (15)
C215—Ni3—C210	173.0 (2)	C201—N1—Sn1	168.7 (4)
C211^ii^—Ni3—C213	174.2 (2)	C202—N2—Sn1	157.4 (4)
C214^iii^—Ni4—C201	179.0 (2)	C203—N3—Sn2	162.0 (4)
C216^iv^—Ni4—C204^v^	177.8 (2)	C204—N4—Sn2	173.5 (4)
C15—Sn1—C1	127.0 (2)	C205—N5—Sn3	169.1 (4)
N2—Sn1—N1	177.2 (2)	C206—N6—Sn3	164.3 (3)
C29—Sn2—C22	121.0 (2)	C207—N7—Sn4	155.8 (4)
N3—Sn2—N4	178.15 (14)	C208—N8—Sn4	152.1 (4)
C50—Sn3—C43	127.12 (19)	C209—N9—Sn5	166.3 (4)
N5—Sn3—N6	177.07 (14)	C210—N10—Sn5	165.8 (4)
C78*A*—Sn4—C71	127.0 (6)	C211—N11—Sn6	155.1 (4)
N7—Sn4—N8	177.0 (2)	C212—N12—Sn6	170.9 (4)
C99—Sn5—C85	123.2 (2)	C213—N13—Sn7	162.0 (4)
N9—Sn5—N10	176.91 (15)	C214—N14—Sn7	154.2 (4)
C106—Sn6—C120	121.3 (2)	C215—N15—Sn8	158.5 (4)
N12—Sn6—N11	173.85 (15)	C216—N16—Sn8	170.3 (4)

Symmetry codes: (i) 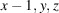; (ii) 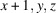; (iii) 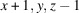; (iv) 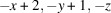; (v) 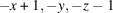.

**Table 2:** Hydrogen-bond geometry (Å, °) *Cg*9, *Cg*13, *Cg*15, *Cg*18, and *Cg*23 are the centroids of rings C58–C63, C86–C91, C100–C105, C121–C126 and C157–C162, respectively.

*D*—H⋯*A*	*D*—H	H⋯*A*	*D*⋯*A*	*D*—H⋯*A*
C117—H11*H*⋯*Cg*9^i^	0.95	2.85	3.77 (2)	163
C165—H16*F*⋯*Cg*23	0.95	2.70	3.505 (7)	142
C40—H40⋯*Cg*13^vi^	0.95	2.63	3.502 (6)	153
C76—H76⋯*Cg*15	0.95	2.78	3.377 (6)	122
C87—H87⋯*Cg*15	0.95	2.97	3.537 (5)	120
C90—H90⋯*Cg*18	0.99	2.68	3.584 (5)	159

Symmetry codes: (i) 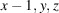; (vi) 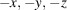.

**Table 3:** Experimental details

Crystal data	
Chemical formula	[NiSn_2_(C_7_H_7_)_6_(CN)_4_]
*M*_r_	946.92
Crystal system, space group	Triclinic, *P* 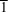
Temperature (K)	173
*a*, *b*, *c* (Å)	14.1316 (5), 21.5593 (9), 28.7274 (9)
α, β, γ (°)	100.478 (3), 90.096 (3), 104.259 (3)
*V* (Å^3^)	8331.3 (5)
*Z*	8
Radiation type	Mo *K*α
μ (mm^−1^)	1.67
Crystal size (mm)	0.45 × 0.40 × 0.10

Data collection	
Diffractometer	Stoe *IPDS* 2
Absorption correction	Multi-scan (*MULABS*; Spek, 2020 ▸)
*T*_min_, *T*_max_	0.594, 1.000
No. of measured, independent and observed [*I* > 2σ(*I*)] reflections	89616, 32732, 23496
*R*_int_	0.075
(sin θ/λ)_max_ (Å^−1^)	0.617

Refinement	
*R*[*F*^2^ > 2σ(*F*^2^)], *wR*(*F*^2^), *S*	0.040, 0.091, 0.94
No. of reflections	32732
No. of parameters	1881
No. of restraints	2
H-atom treatment	H-atom parameters constrained
Δρ_max_, Δρ_min_ (e Å^−3^)	0.97, −1.50

Computer programs: *X-AREA* and *X-RED32* (Stoe & Cie, 2002 ▸), *SHELXT2019/3* (Sheldrick, 2015*a* ▸), *SHELXL2019/3* (Sheldrick, 2015*b* ▸), *PLATON* (Spek, 2020 ▸), *Mercury* (Macrae *et al.*, 2020 ▸) and *publCIF* (Westrip, 2010 ▸).

## supplementary crystallographic information

### Poly[hexabenzyltetra-µ-cyanato-nickel(II)ditin(IV)] Crystal data

**Table:**

[NiSn_2_(C_7_H_7_)_6_(CN)_4_]	*Z* = 8
*M**_r_* = 946.92	*F*(000) = 3792
Triclinic, *P*1	*D*_x_ = 1.510 Mg m^−^^3^
*a* = 14.1316 (5) Å	Mo *K*α radiation, λ = 0.71073 Å
*b* = 21.5593 (9) Å	Cell parameters from 70477 reflections
*c* = 28.7274 (9) Å	θ = 1.6–29.6°
α = 100.478 (3)°	µ = 1.67 mm^−^^1^
β = 90.096 (3)°	*T* = 173 K
γ = 104.259 (3)°	Plate, colourless
*V* = 8331.3 (5) Å^3^	0.45 × 0.40 × 0.10 mm

### Poly[hexabenzyltetra-µ-cyanato-nickel(II)ditin(IV)] Data collection

**Table:**

Stoe IPDS 2 diffractometer	32732 independent reflections
Radiation source: fine-focus sealed tube	23496 reflections with *I* > 2σ(*I*)
Plane graphite monochromator	*R*_int_ = 0.075
φ + ω scans	θ_max_ = 26.0°, θ_min_ = 1.6°
Absorption correction: multi-scan (MULABS; Spek, 2020)	*h* = −17→17
*T*_min_ = 0.594, *T*_max_ = 1.000	*k* = −25→26
89616 measured reflections	*l* = −35→35

### Poly[hexabenzyltetra-µ-cyanato-nickel(II)ditin(IV)] Refinement

**Table:**

Refinement on *F*^2^	Primary atom site location: dual
Least-squares matrix: full	Secondary atom site location: difference Fourier map
*R*[*F*^2^ > 2σ(*F*^2^)] = 0.040	Hydrogen site location: inferred from neighbouring sites
*wR*(*F*^2^) = 0.091	H-atom parameters constrained
*S* = 0.94	*w* = 1/[σ^2^(*F*_o_^2^) + (0.0426*P*)^2^] where *P* = (*F*_o_^2^ + 2*F*_c_^2^)/3
32732 reflections	(Δ/σ)_max_ = 0.002
1881 parameters	Δρ_max_ = 0.97 e Å^−^^3^
2 restraints	Δρ_min_ = −1.50 e Å^−^^3^

### Poly[hexabenzyltetra-µ-cyanato-nickel(II)ditin(IV)] Special details

**Table:**

Geometry. All esds (except the esd in the dihedral angle between two l.s. planes) are estimated using the full covariance matrix. The cell esds are taken into account individually in the estimation of esds in distances, angles and torsion angles; correlations between esds in cell parameters are only used when they are defined by crystal symmetry. An approximate (isotropic) treatment of cell esds is used for estimating esds involving l.s. planes.

### Poly[hexabenzyltetra-µ-cyanato-nickel(II)ditin(IV)] Fractional atomic coordinates and isotropic or equivalent isotropic displacement parameters (Å^2^)

**Table:**

	*x*	*y*	*z*	*U*_iso_*/*U*_eq_	Occ. (<1)
Sn1	0.74176 (2)	0.16723 (2)	−0.39930 (2)	0.02748 (7)	
Sn2	0.22307 (2)	−0.01331 (2)	−0.36481 (2)	0.02741 (7)	
Sn3	0.27227 (2)	0.20171 (2)	−0.12600 (2)	0.02383 (7)	
Sn4	0.77981 (2)	0.22979 (2)	−0.12461 (2)	0.04000 (9)	
Sn5	0.35957 (2)	0.31398 (2)	0.12241 (2)	0.02655 (7)	
Sn6	−0.12507 (2)	0.35535 (2)	0.16204 (2)	0.02573 (7)	
Sn7	0.28873 (2)	0.30653 (2)	0.36291 (2)	0.02792 (7)	
Sn8	0.79539 (2)	0.55483 (2)	0.41363 (2)	0.02919 (7)	
Ni1	0.51107 (3)	0.14701 (3)	−0.26043 (2)	0.02364 (12)	
Ni2	0.04780 (3)	0.27176 (3)	0.00903 (2)	0.02308 (12)	
Ni3	0.59957 (3)	0.39166 (3)	0.27454 (2)	0.02267 (12)	
Ni4	1.01007 (3)	0.22333 (3)	−0.52626 (2)	0.02371 (12)	
N1	0.8724 (3)	0.2021 (2)	−0.44791 (15)	0.0385 (10)	
N2	0.6216 (3)	0.1364 (2)	−0.34951 (15)	0.0358 (9)	
N3	0.3279 (3)	0.07183 (19)	−0.31731 (15)	0.0348 (9)	
N4	0.1176 (3)	−0.1010 (2)	−0.41616 (15)	0.0410 (11)	
N5	0.3893 (3)	0.1763 (2)	−0.17760 (14)	0.0345 (9)	
N6	0.1525 (2)	0.23008 (19)	−0.07698 (14)	0.0300 (8)	
N7	0.6947 (3)	0.1971 (3)	−0.19730 (16)	0.0598 (16)	
N8	0.8649 (3)	0.2572 (2)	−0.05072 (15)	0.0405 (10)	
N9	0.2341 (2)	0.2879 (2)	0.06545 (14)	0.0347 (9)	
N10	0.4819 (3)	0.3435 (2)	0.18284 (15)	0.0392 (10)	
N11	−0.2197 (3)	0.3988 (2)	0.21979 (14)	0.0355 (9)	
N12	−0.0426 (3)	0.3153 (2)	0.09948 (14)	0.0355 (10)	
N13	0.4197 (3)	0.3634 (2)	0.33032 (16)	0.0373 (10)	
N14	0.1514 (3)	0.2436 (2)	0.39656 (15)	0.0410 (11)	
N15	0.7231 (3)	0.4651 (2)	0.36094 (15)	0.0371 (10)	
N16	0.8713 (3)	0.6506 (2)	0.46964 (15)	0.0388 (10)	
C1	0.8409 (3)	0.1374 (3)	−0.35625 (19)	0.0405 (12)	
H1A	0.908807	0.162497	−0.359006	0.049*	
H1B	0.824405	0.145576	−0.322567	0.049*	
C2	0.8317 (4)	0.0680 (3)	−0.3729 (2)	0.0446 (13)	
C3	0.8934 (4)	0.0465 (4)	−0.4055 (2)	0.0609 (17)	
H3	0.945055	0.077049	−0.416421	0.073*	
C4	0.8798 (6)	−0.0206 (5)	−0.4228 (3)	0.082 (2)	
H4	0.921901	−0.035693	−0.445415	0.098*	
C5	0.8045 (7)	−0.0644 (4)	−0.4064 (3)	0.085 (3)	
H5	0.794724	−0.109782	−0.417782	0.102*	
C6	0.7462 (6)	−0.0433 (4)	−0.3749 (4)	0.082 (3)	
H6	0.694570	−0.073800	−0.364063	0.098*	
C7	0.7588 (4)	0.0213 (3)	−0.3578 (3)	0.0592 (17)	
H7	0.716134	0.034906	−0.334843	0.071*	
C8	0.6785 (3)	0.0898 (2)	−0.45689 (17)	0.0346 (11)	
H8A	0.731858	0.075526	−0.474569	0.042*	
H8B	0.640367	0.052368	−0.443747	0.042*	
C9	0.6491 (3)	0.1396 (3)	−0.52673 (19)	0.0401 (12)	
H9	0.717766	0.152244	−0.529844	0.048*	
C10	0.5875 (4)	0.1542 (3)	−0.5580 (2)	0.0466 (13)	
H10	0.614033	0.177572	−0.581989	0.056*	
C11	0.4877 (4)	0.1350 (3)	−0.5548 (2)	0.0522 (15)	
H11	0.445289	0.144668	−0.576602	0.063*	
C12	0.4504 (4)	0.1021 (4)	−0.5201 (2)	0.0631 (19)	
H12	0.381641	0.088467	−0.517777	0.076*	
C13	0.5119 (4)	0.0883 (3)	−0.4880 (2)	0.0530 (16)	
H13	0.484894	0.065834	−0.463694	0.064*	
C14	0.6129 (3)	0.1069 (2)	−0.49103 (16)	0.0292 (10)	
C15	0.7189 (4)	0.2630 (3)	−0.3935 (2)	0.0447 (13)	
H15A	0.783303	0.294334	−0.393320	0.054*	
H15B	0.680636	0.264089	−0.422092	0.054*	
C16	0.6680 (4)	0.2857 (3)	−0.35123 (19)	0.0392 (12)	
C17	0.5688 (4)	0.2807 (3)	−0.3525 (3)	0.0599 (17)	
H17	0.531877	0.261553	−0.381502	0.072*	
C18	0.5220 (6)	0.3021 (5)	−0.3140 (4)	0.093 (3)	
H18	0.454325	0.300153	−0.316819	0.111*	
C19	0.5711 (8)	0.3259 (4)	−0.2722 (4)	0.098 (3)	
H19	0.537587	0.338709	−0.244866	0.117*	
C20	0.6684 (8)	0.3316 (4)	−0.2687 (3)	0.088 (3)	
H20	0.702985	0.349138	−0.238925	0.106*	
C21	0.7187 (5)	0.3123 (3)	−0.3080 (2)	0.0597 (16)	
H21	0.787291	0.317270	−0.305280	0.072*	
C22	0.2884 (4)	0.0233 (2)	−0.42448 (18)	0.0391 (12)	
H22A	0.360439	0.033620	−0.419953	0.047*	
H22B	0.268773	−0.010754	−0.453343	0.047*	
C23	0.2585 (4)	0.0829 (3)	−0.43110 (18)	0.0418 (12)	
C24	0.3179 (4)	0.1442 (3)	−0.4150 (2)	0.0480 (14)	
H24	0.379486	0.148684	−0.399590	0.058*	
C25	0.2895 (6)	0.1995 (3)	−0.4208 (2)	0.0641 (18)	
H25	0.331339	0.241372	−0.409311	0.077*	
C26	0.2005 (7)	0.1937 (4)	−0.4431 (3)	0.081 (2)	
H26	0.180643	0.231407	−0.447427	0.097*	
C27	0.1424 (6)	0.1340 (4)	−0.4587 (3)	0.085 (2)	
H27	0.080984	0.129938	−0.473953	0.102*	
C28	0.1690 (5)	0.0782 (3)	−0.4532 (2)	0.0628 (18)	
H28	0.126016	0.036665	−0.464523	0.075*	
C29	0.0968 (3)	0.0136 (2)	−0.33590 (18)	0.0350 (11)	
H29A	0.115376	0.059595	−0.319213	0.042*	
H29B	0.048367	0.009738	−0.361857	0.042*	
C30	0.3512 (4)	−0.0606 (3)	−0.29896 (19)	0.0390 (12)	
C31	0.3282 (4)	−0.0470 (3)	−0.2524 (2)	0.0517 (15)	
H31	0.261465	−0.057542	−0.244722	0.062*	
C32	0.3981 (4)	−0.0189 (4)	−0.2168 (2)	0.0636 (18)	
H32	0.379678	−0.009275	−0.185063	0.076*	
C33	0.4953 (4)	−0.0044 (3)	−0.2271 (2)	0.0611 (17)	
H33	0.544402	0.015061	−0.202525	0.073*	
C34	0.5203 (4)	−0.0181 (3)	−0.2727 (2)	0.0575 (16)	
H34	0.587288	−0.008732	−0.279941	0.069*	
C35	0.4492 (4)	−0.0458 (3)	−0.3088 (2)	0.0495 (14)	
H35	0.467906	−0.054606	−0.340547	0.059*	
C36	0.2733 (4)	−0.0862 (3)	−0.33744 (19)	0.0417 (12)	
H36A	0.216780	−0.115024	−0.325300	0.050*	
H36B	0.298210	−0.113206	−0.363853	0.050*	
C37	0.0521 (3)	−0.0292 (2)	−0.30199 (16)	0.0289 (10)	
C38	−0.0192 (3)	−0.0869 (2)	−0.31690 (18)	0.0336 (11)	
H38	−0.041640	−0.099340	−0.349240	0.040*	
C39	−0.0574 (3)	−0.1262 (3)	−0.28529 (19)	0.0401 (12)	
H39	−0.105549	−0.165667	−0.296094	0.048*	
C40	−0.0269 (3)	−0.1089 (3)	−0.23823 (19)	0.0409 (12)	
H40	−0.053275	−0.136411	−0.216599	0.049*	
C41	0.0427 (4)	−0.0511 (3)	−0.22272 (19)	0.0434 (13)	
H41	0.063245	−0.038088	−0.190138	0.052*	
C42	0.0820 (3)	−0.0125 (3)	−0.25447 (19)	0.0418 (13)	
H42	0.130970	0.026557	−0.243583	0.050*	
C43	0.2501 (4)	0.2695 (2)	−0.16813 (18)	0.0369 (11)	
H43A	0.204267	0.293645	−0.152134	0.044*	
H43B	0.313208	0.301586	−0.169422	0.044*	
C44	0.2108 (2)	0.24009 (18)	−0.21778 (9)	0.0304 (10)	
C45	0.1136 (2)	0.2056 (2)	−0.22681 (12)	0.0567 (17)	
H45	0.071947	0.199292	−0.201256	0.068*	
C46	0.0774 (2)	0.1804 (2)	−0.27322 (15)	0.080 (2)	
H46	0.010944	0.156889	−0.279387	0.095*	
C47	0.1383 (4)	0.1897 (2)	−0.31060 (10)	0.074 (2)	
H47	0.113580	0.172496	−0.342325	0.089*	
C48	0.2356 (3)	0.2242 (2)	−0.30158 (10)	0.074 (2)	
H48	0.277219	0.230506	−0.327133	0.089*	
C49	0.2718 (2)	0.2494 (2)	−0.25517 (13)	0.0566 (16)	
H49	0.338223	0.272909	−0.249002	0.068*	
C50	0.2000 (3)	0.1005 (2)	−0.13539 (17)	0.0330 (10)	
H50A	0.161637	0.086874	−0.166014	0.040*	
H50B	0.249391	0.074792	−0.136404	0.040*	
C51	0.1330 (3)	0.0865 (2)	−0.09593 (17)	0.0313 (10)	
C52	0.0331 (3)	0.0761 (2)	−0.10287 (19)	0.0397 (12)	
H52	0.005576	0.072983	−0.133656	0.048*	
C53	−0.0274 (4)	0.0700 (3)	−0.0649 (2)	0.0438 (13)	
H53	−0.095982	0.062695	−0.070016	0.053*	
C54	0.0110 (4)	0.0746 (3)	−0.0202 (2)	0.0485 (14)	
H54	−0.030743	0.071284	0.005598	0.058*	
C55	0.1101 (4)	0.0840 (3)	−0.0129 (2)	0.0459 (13)	
H55	0.137454	0.087216	0.017961	0.055*	
C56	0.1698 (3)	0.0888 (3)	−0.05105 (18)	0.0380 (12)	
H56	0.237925	0.093781	−0.046081	0.046*	
C57	0.3777 (3)	0.2430 (2)	−0.06813 (17)	0.0307 (10)	
H57A	0.347881	0.233948	−0.038126	0.037*	
H57B	0.433785	0.223048	−0.072653	0.037*	
C58	0.4120 (3)	0.3145 (2)	−0.06545 (17)	0.0305 (10)	
C59	0.4808 (3)	0.3402 (3)	−0.0958 (2)	0.0439 (13)	
H59	0.507761	0.311787	−0.118043	0.053*	
C60	0.5108 (4)	0.4069 (3)	−0.0939 (3)	0.0629 (18)	
H60	0.558536	0.424128	−0.114554	0.076*	
C61	0.4715 (5)	0.4476 (3)	−0.0623 (3)	0.077 (2)	
H61	0.493424	0.493373	−0.060511	0.092*	
C62	0.4002 (5)	0.4234 (3)	−0.0329 (3)	0.070 (2)	
H62	0.371134	0.451922	−0.011852	0.084*	
C63	0.3721 (4)	0.3569 (3)	−0.0347 (2)	0.0453 (13)	
H63	0.323963	0.339959	−0.014162	0.054*	
C64A	0.8373 (16)	0.1437 (13)	−0.1274 (4)	0.065 (6)	0.69 (4)
H64A	0.908877	0.158951	−0.120767	0.078*	0.69 (4)
H64B	0.809679	0.121806	−0.101181	0.078*	0.69 (4)
C64B	0.8701 (15)	0.1727 (15)	−0.1415 (17)	0.051 (10)	0.31 (4)
H64C	0.920239	0.193808	−0.161580	0.061*	0.31 (4)
H64D	0.904255	0.170499	−0.111947	0.061*	0.31 (4)
C65	0.8200 (3)	0.0955 (3)	−0.17014 (13)	0.069 (2)	
C66	0.7492 (3)	0.0412 (4)	−0.16283 (17)	0.095 (3)	
H66	0.714643	0.042809	−0.134469	0.114*	
C67	0.7289 (3)	−0.0156 (3)	−0.1970 (3)	0.103 (3)	
H67	0.680434	−0.052715	−0.192003	0.123*	
C68	0.7794 (4)	−0.0180 (2)	−0.2385 (2)	0.087 (3)	
H68	0.765518	−0.056747	−0.261850	0.104*	
C69	0.8503 (3)	0.0364 (3)	−0.24581 (13)	0.082 (3)	
H69	0.884811	0.034745	−0.274164	0.098*	
C70	0.8706 (3)	0.0931 (2)	−0.21163 (16)	0.070 (2)	
H70	0.919020	0.130270	−0.216631	0.084*	
C71	0.6430 (3)	0.2286 (3)	−0.09325 (19)	0.0418 (13)	
H71A	0.653544	0.265929	−0.066398	0.050*	
H71B	0.598374	0.236255	−0.116945	0.050*	
C72	0.5923 (3)	0.1693 (3)	−0.07555 (17)	0.0361 (12)	
C73	0.5328 (4)	0.1166 (3)	−0.10516 (19)	0.0433 (13)	
H73	0.527785	0.116937	−0.138092	0.052*	
C74	0.4802 (4)	0.0631 (3)	−0.0879 (2)	0.0501 (14)	
H74	0.438151	0.027809	−0.108731	0.060*	
C75	0.4888 (4)	0.0611 (3)	−0.0404 (2)	0.0516 (15)	
H75	0.452841	0.024602	−0.028276	0.062*	
C76	0.5500 (4)	0.1126 (4)	−0.0109 (2)	0.0554 (16)	
H76	0.557126	0.111383	0.021753	0.066*	
C77	0.6007 (4)	0.1657 (3)	−0.02806 (19)	0.0516 (16)	
H77	0.642737	0.200782	−0.006990	0.062*	
C78A	0.8639 (11)	0.3029 (8)	−0.1560 (6)	0.066 (4)	0.510 (12)
H78A	0.892769	0.282368	−0.184246	0.079*	0.510 (12)
H78B	0.820423	0.327444	−0.167083	0.079*	0.510 (12)
C79A	0.9444 (6)	0.3500 (5)	−0.1249 (4)	0.056 (5)	0.510 (12)
C80A	1.0406 (7)	0.3451 (5)	−0.1245 (3)	0.065 (2)	0.510 (12)
H80A	1.059989	0.313772	−0.147706	0.078*	0.510 (12)
C81A	1.1086 (5)	0.3862 (5)	−0.0904 (4)	0.065 (2)	0.510 (12)
H81A	1.174404	0.382853	−0.090148	0.078*	0.510 (12)
C82A	1.0803 (7)	0.4321 (5)	−0.0565 (3)	0.065 (2)	0.510 (12)
H82A	1.126761	0.460104	−0.033109	0.078*	0.510 (12)
C83A	0.9840 (7)	0.4369 (5)	−0.0568 (3)	0.065 (2)	0.510 (12)
H83A	0.964703	0.468274	−0.033627	0.078*	0.510 (12)
C84A	0.9161 (5)	0.3959 (5)	−0.0910 (4)	0.065 (2)	0.510 (12)
H84A	0.850286	0.399193	−0.091185	0.078*	0.510 (12)
C78B	0.8431 (11)	0.3335 (8)	−0.1346 (7)	0.066 (4)	0.490 (12)
H78C	0.845508	0.333874	−0.169033	0.079*	0.490 (12)
H78D	0.798408	0.360387	−0.121357	0.079*	0.490 (12)
C79B	0.9430 (6)	0.3645 (5)	−0.1122 (4)	0.032 (3)	0.490 (12)
C80B	1.0304 (8)	0.3604 (6)	−0.1331 (3)	0.086 (3)	0.490 (12)
H80B	1.029678	0.334978	−0.163854	0.104*	0.490 (12)
C81B	1.1190 (6)	0.3933 (6)	−0.1089 (5)	0.086 (3)	0.490 (12)
H81B	1.178731	0.390497	−0.123185	0.104*	0.490 (12)
C82B	1.1201 (7)	0.4305 (5)	−0.0639 (4)	0.086 (3)	0.490 (12)
H82B	1.180583	0.453009	−0.047368	0.104*	0.490 (12)
C83B	1.0326 (10)	0.4346 (5)	−0.0430 (4)	0.086 (3)	0.490 (12)
H83B	1.033383	0.460004	−0.012221	0.104*	0.490 (12)
C84B	0.9441 (7)	0.4016 (5)	−0.0672 (5)	0.086 (3)	0.490 (12)
H84B	0.884329	0.404486	−0.052890	0.104*	0.490 (12)
C85	0.2663 (3)	0.2722 (3)	0.17329 (17)	0.0354 (11)	
H85A	0.307225	0.261131	0.196983	0.042*	
H85B	0.234787	0.305476	0.190155	0.042*	
C86	0.1876 (3)	0.2120 (2)	0.15304 (16)	0.0302 (10)	
C87	0.2037 (3)	0.1515 (2)	0.14668 (17)	0.0314 (10)	
H87	0.265698	0.146823	0.156042	0.038*	
C88	0.1318 (3)	0.0965 (2)	0.12684 (18)	0.0357 (11)	
H88	0.144459	0.054584	0.122829	0.043*	
C89	0.0414 (3)	0.1030 (2)	0.11290 (17)	0.0347 (11)	
H89	−0.008245	0.065685	0.098937	0.042*	
C90	0.0244 (3)	0.1636 (2)	0.11946 (18)	0.0353 (11)	
H90	−0.037585	0.168374	0.110088	0.042*	
C91	0.0961 (3)	0.2180 (2)	0.13953 (17)	0.0328 (11)	
H91	0.083071	0.259782	0.144156	0.039*	
C92	0.3801 (3)	0.4137 (2)	0.1178 (2)	0.0392 (12)	
H92A	0.441842	0.438862	0.135599	0.047*	
H92B	0.387884	0.417822	0.084216	0.047*	
C93	0.2999 (3)	0.4439 (3)	0.1363 (2)	0.0429 (13)	
C94	0.2311 (4)	0.4515 (3)	0.1042 (3)	0.068 (2)	
H94	0.234817	0.436587	0.071211	0.082*	
C95	0.1575 (5)	0.4808 (4)	0.1205 (4)	0.088 (3)	
H95	0.112143	0.486945	0.098354	0.106*	
C96	0.1496 (5)	0.5004 (4)	0.1662 (5)	0.090 (3)	
H96	0.097549	0.519379	0.176546	0.108*	
C97	0.2150 (6)	0.4938 (4)	0.1989 (4)	0.088 (3)	
H97	0.208858	0.508281	0.231729	0.106*	
C98	0.2920 (5)	0.4652 (3)	0.1832 (3)	0.0632 (17)	
H98	0.338347	0.460903	0.205555	0.076*	
C99	0.4460 (3)	0.2621 (3)	0.07734 (17)	0.0343 (11)	
H99A	0.432452	0.264952	0.044078	0.041*	
H99B	0.516014	0.283416	0.085441	0.041*	
C100	0.4276 (3)	0.1933 (3)	0.08107 (16)	0.0320 (10)	
C101	0.4832 (4)	0.1721 (3)	0.1124 (2)	0.0446 (13)	
H10A	0.533503	0.202868	0.132341	0.053*	
C102	0.4656 (4)	0.1075 (4)	0.1145 (2)	0.0587 (17)	
H10B	0.503833	0.094083	0.136119	0.070*	
C103	0.3936 (5)	0.0612 (3)	0.0859 (3)	0.0628 (19)	
H10C	0.382993	0.016335	0.087189	0.075*	
C104	0.3381 (4)	0.0813 (3)	0.0557 (2)	0.0511 (15)	
H10D	0.287700	0.050036	0.036107	0.061*	
C105	0.3539 (3)	0.1455 (3)	0.05315 (18)	0.0382 (12)	
H10E	0.313796	0.158151	0.031920	0.046*	
C106	−0.0140 (3)	0.3620 (3)	0.21552 (17)	0.0355 (11)	
H10F	0.038761	0.343856	0.200459	0.043*	
H10G	0.014603	0.408450	0.229338	0.043*	
C107	−0.0526 (3)	0.3263 (2)	0.25450 (17)	0.0319 (10)	
C108	−0.0671 (3)	0.2605 (3)	0.2492 (2)	0.0439 (13)	
H10H	−0.052936	0.236257	0.220187	0.053*	
C109	−0.1022 (4)	0.2285 (3)	0.2858 (2)	0.0518 (15)	
H10I	−0.111320	0.182643	0.281788	0.062*	
C110	−0.1242 (4)	0.2631 (3)	0.3281 (2)	0.0462 (13)	
H11A	−0.148838	0.241157	0.353056	0.055*	
C111	−0.1103 (4)	0.3283 (3)	0.3337 (2)	0.0489 (14)	
H11B	−0.124753	0.352423	0.362691	0.059*	
C112	−0.0746 (4)	0.3604 (3)	0.29648 (19)	0.0434 (12)	
H11C	−0.065546	0.406248	0.300455	0.052*	
C113	−0.1231 (3)	0.4364 (2)	0.12819 (17)	0.0328 (10)	
H11D	−0.128391	0.474346	0.152313	0.039*	
H11E	−0.060198	0.448312	0.112864	0.039*	
C114	−0.2051 (3)	0.4204 (2)	0.09194 (18)	0.0330 (11)	
C115	−0.1923 (4)	0.3962 (3)	0.0453 (2)	0.0495 (14)	
H11F	−0.129644	0.390465	0.036772	0.059*	
C116	−0.2662 (5)	0.3798 (4)	0.0103 (3)	0.0688 (19)	
H11G	−0.254639	0.363581	−0.021619	0.083*	
C117	−0.3556 (5)	0.3876 (4)	0.0229 (4)	0.089 (3)	
H11H	−0.407458	0.376246	−0.000641	0.107*	
C118	−0.3724 (4)	0.4111 (5)	0.0680 (3)	0.085 (3)	
H11I	−0.435731	0.416040	0.075848	0.102*	
C119	−0.2960 (4)	0.4287 (4)	0.1042 (2)	0.0613 (18)	
H11J	−0.307667	0.445767	0.135935	0.074*	
C120	−0.2507 (3)	0.2726 (3)	0.1475 (2)	0.0422 (13)	
H12A	−0.280302	0.266879	0.178091	0.051*	
H12B	−0.298984	0.284568	0.128049	0.051*	
C121	−0.2392 (3)	0.2085 (2)	0.12352 (19)	0.0374 (12)	
C122	−0.2308 (3)	0.1941 (3)	0.0749 (2)	0.0474 (14)	
H12C	−0.229254	0.226880	0.056647	0.057*	
C123	−0.2246 (4)	0.1331 (3)	0.0526 (3)	0.0611 (18)	
H12D	−0.219746	0.124480	0.019228	0.073*	
C124	−0.2254 (4)	0.0855 (3)	0.0773 (3)	0.065 (2)	
H12E	−0.220671	0.043656	0.061581	0.078*	
C125	−0.2331 (4)	0.0984 (3)	0.1258 (3)	0.0586 (18)	
H12F	−0.233467	0.065284	0.143658	0.070*	
C126	−0.2401 (3)	0.1589 (3)	0.1486 (2)	0.0446 (13)	
H12G	−0.245687	0.166959	0.181945	0.053*	
C127	0.3163 (3)	0.3698 (2)	0.43085 (17)	0.0332 (11)	
H12H	0.339055	0.415078	0.425420	0.040*	
H12I	0.253073	0.366482	0.446239	0.040*	
C128	0.3880 (3)	0.3598 (2)	0.46540 (18)	0.0353 (11)	
C129	0.3809 (5)	0.3007 (3)	0.4788 (2)	0.0564 (16)	
H12J	0.329537	0.264318	0.465114	0.068*	
C130	0.4469 (5)	0.2929 (4)	0.5117 (2)	0.0637 (18)	
H13A	0.441804	0.251263	0.519558	0.076*	
C131	0.5199 (4)	0.3456 (4)	0.5329 (2)	0.0622 (18)	
H13B	0.564392	0.340967	0.556055	0.075*	
C132	0.5278 (4)	0.4042 (4)	0.5203 (3)	0.0659 (19)	
H13C	0.577842	0.440791	0.534853	0.079*	
C133	0.4633 (4)	0.4110 (3)	0.4865 (2)	0.0502 (14)	
H13D	0.471312	0.452207	0.477495	0.060*	
C134	0.1909 (4)	0.3363 (3)	0.3192 (2)	0.0458 (13)	
H13E	0.211926	0.332062	0.286175	0.055*	
H13F	0.123748	0.308418	0.319222	0.055*	
C135	0.1930 (3)	0.4041 (3)	0.3387 (2)	0.0442 (13)	
C136	0.2563 (5)	0.4556 (4)	0.3233 (3)	0.079 (2)	
H13G	0.295219	0.446764	0.297138	0.095*	
C137	0.2643 (7)	0.5199 (4)	0.3451 (5)	0.113 (4)	
H13H	0.309829	0.554126	0.334448	0.135*	
C138	0.2082 (7)	0.5338 (5)	0.3812 (4)	0.104 (3)	
H13I	0.213003	0.577920	0.395418	0.125*	
C139	0.1443 (6)	0.4848 (4)	0.3975 (3)	0.079 (2)	
H13J	0.105288	0.494503	0.423397	0.095*	
C140	0.1372 (4)	0.4218 (4)	0.3762 (2)	0.0555 (16)	
H14A	0.091789	0.388189	0.387655	0.067*	
C141	0.3355 (3)	0.2179 (3)	0.3487 (2)	0.0416 (12)	
H14B	0.341474	0.203558	0.379222	0.050*	
H14C	0.283483	0.184068	0.328907	0.050*	
C142	0.4297 (3)	0.2203 (2)	0.32462 (19)	0.0376 (11)	
C143	0.4334 (5)	0.1988 (3)	0.2770 (2)	0.0599 (16)	
H14D	0.374797	0.180502	0.257825	0.072*	
C144	0.5265 (6)	0.2043 (4)	0.2562 (3)	0.075 (2)	
H14E	0.530212	0.189223	0.223215	0.090*	
C145	0.6093 (5)	0.2312 (4)	0.2840 (3)	0.071 (2)	
H14F	0.670900	0.235197	0.270090	0.085*	
C146	0.6063 (4)	0.2522 (4)	0.3306 (3)	0.0666 (19)	
H14G	0.665326	0.270936	0.349346	0.080*	
C147	0.5188 (4)	0.2469 (3)	0.3510 (2)	0.0500 (14)	
H14H	0.517759	0.261589	0.384220	0.060*	
C148	0.7481 (4)	0.5028 (3)	0.46960 (19)	0.0406 (12)	
H14I	0.772124	0.531704	0.500380	0.049*	
H14J	0.675738	0.490958	0.468971	0.049*	
C149	0.7830 (4)	0.4430 (3)	0.46599 (18)	0.0383 (11)	
C151	0.7209 (5)	0.3820 (3)	0.4530 (2)	0.0594 (16)	
H15C	0.654035	0.377783	0.445018	0.071*	
C152	0.7549 (7)	0.3264 (3)	0.4513 (3)	0.081 (2)	
H15D	0.710941	0.284528	0.443047	0.097*	
C153	0.8517 (7)	0.3317 (4)	0.4617 (3)	0.082 (2)	
H15E	0.874894	0.293689	0.460531	0.099*	
C154	0.9137 (6)	0.3913 (4)	0.4735 (3)	0.072 (2)	
H15F	0.980791	0.395374	0.480689	0.087*	
C155	0.8801 (4)	0.4457 (3)	0.4750 (2)	0.0573 (16)	
H15G	0.925331	0.487116	0.482673	0.069*	
C156	0.9374 (3)	0.5537 (3)	0.38686 (19)	0.0424 (13)	
H15H	0.988260	0.576062	0.412223	0.051*	
H15I	0.941877	0.508043	0.377555	0.051*	
C157	0.9556 (3)	0.5863 (3)	0.3455 (2)	0.0398 (12)	
C158	0.9886 (4)	0.6527 (3)	0.3505 (3)	0.0624 (18)	
H15K	1.002140	0.678634	0.381350	0.075*	
C159	1.0024 (5)	0.6828 (4)	0.3115 (4)	0.085 (3)	
H15L	1.025438	0.728896	0.316061	0.102*	
C160	0.9836 (6)	0.6476 (5)	0.2670 (4)	0.090 (3)	
H16A	0.993275	0.668312	0.240323	0.108*	
C161	0.9504 (6)	0.5814 (4)	0.2614 (3)	0.078 (2)	
H16B	0.936638	0.555955	0.230413	0.094*	
C162	0.9366 (4)	0.5512 (3)	0.2996 (2)	0.0571 (16)	
H16C	0.913561	0.505091	0.294629	0.069*	
C163	0.7137 (4)	0.6155 (3)	0.3888 (2)	0.0458 (13)	
H16D	0.662644	0.621524	0.411517	0.055*	
H16E	0.758421	0.658917	0.389203	0.055*	
C164	0.6658 (3)	0.5910 (2)	0.3406 (2)	0.0417 (13)	
C165	0.7173 (4)	0.6031 (3)	0.3008 (2)	0.0547 (16)	
H16F	0.783139	0.628241	0.304785	0.066*	
C166	0.6755 (5)	0.5795 (4)	0.2554 (3)	0.072 (2)	
H16G	0.712474	0.588098	0.228717	0.087*	
C167	0.5793 (5)	0.5432 (4)	0.2493 (3)	0.072 (2)	
H16H	0.550221	0.525603	0.218355	0.087*	
C168	0.5269 (5)	0.5330 (3)	0.2881 (3)	0.066 (2)	
H16I	0.459976	0.509839	0.283972	0.079*	
C169	0.5685 (4)	0.5555 (3)	0.3332 (3)	0.0586 (17)	
H16J	0.530550	0.546785	0.359613	0.070*	
C201	0.9262 (3)	0.2115 (2)	−0.47722 (17)	0.0287 (10)	
C202	0.5802 (3)	0.1384 (2)	−0.31495 (17)	0.0299 (10)	
C203	0.3969 (3)	0.1042 (2)	−0.29595 (16)	0.0274 (10)	
C204	0.0671 (3)	−0.1471 (2)	−0.43789 (17)	0.0327 (11)	
C205	0.4368 (3)	0.1640 (2)	−0.20874 (16)	0.0278 (10)	
C206	0.1104 (3)	0.2458 (2)	−0.04536 (15)	0.0243 (9)	
C207	0.6261 (3)	0.1800 (3)	−0.22266 (17)	0.0369 (12)	
C208	0.9339 (3)	0.2631 (2)	−0.02675 (16)	0.0320 (10)	
C209	0.1627 (3)	0.2817 (2)	0.04404 (15)	0.0274 (10)	
C210	0.5256 (3)	0.3592 (2)	0.21794 (17)	0.0300 (10)	
C211	−0.2884 (3)	0.3990 (2)	0.24082 (15)	0.0262 (9)	
C212	−0.0087 (3)	0.2989 (2)	0.06486 (17)	0.0294 (10)	
C213	0.4881 (3)	0.3760 (2)	0.30919 (16)	0.0274 (10)	
C214	0.0949 (3)	0.2345 (2)	0.42533 (17)	0.0306 (10)	
C215	0.6759 (3)	0.4336 (2)	0.32852 (16)	0.0260 (9)	
C216	0.9158 (3)	0.6988 (2)	0.49134 (16)	0.0286 (10)	

### Poly[hexabenzyltetra-µ-cyanato-nickel(II)ditin(IV)] Atomic displacement parameters (Å^2^)

**Table:**

	*U*^11^	*U*^22^	*U*^33^	*U*^12^	*U*^13^	*U*^23^
Sn1	0.02814 (14)	0.03222 (17)	0.02134 (16)	0.00620 (12)	0.00817 (11)	0.00495 (13)
Sn2	0.02557 (14)	0.02762 (16)	0.02340 (16)	−0.00310 (12)	0.00150 (11)	0.00377 (13)
Sn3	0.02325 (13)	0.02843 (16)	0.01979 (15)	0.00726 (11)	0.00564 (11)	0.00336 (12)
Sn4	0.02049 (14)	0.0667 (3)	0.03389 (19)	0.00723 (14)	−0.00666 (13)	0.01724 (18)
Sn5	0.01812 (12)	0.03399 (17)	0.02558 (16)	0.00274 (11)	−0.00371 (11)	0.00582 (13)
Sn6	0.01925 (12)	0.03435 (17)	0.02370 (16)	0.00855 (12)	0.00510 (11)	0.00320 (13)
Sn7	0.02146 (13)	0.03109 (17)	0.02851 (17)	0.00246 (12)	0.00753 (11)	0.00426 (13)
Sn8	0.02480 (14)	0.02928 (16)	0.02932 (17)	0.00616 (12)	−0.00503 (12)	−0.00428 (13)
Ni1	0.0176 (2)	0.0361 (3)	0.0170 (3)	0.0069 (2)	0.00289 (19)	0.0042 (2)
Ni2	0.0153 (2)	0.0357 (3)	0.0179 (3)	0.0086 (2)	0.00035 (19)	0.0013 (2)
Ni3	0.0149 (2)	0.0306 (3)	0.0196 (3)	0.0037 (2)	0.00064 (19)	0.0001 (2)
Ni4	0.0188 (2)	0.0249 (3)	0.0221 (3)	−0.0023 (2)	0.0037 (2)	0.0014 (2)
N1	0.035 (2)	0.043 (2)	0.036 (2)	0.0048 (18)	0.0167 (18)	0.010 (2)
N2	0.037 (2)	0.038 (2)	0.031 (2)	0.0073 (17)	0.0161 (18)	0.0069 (19)
N3	0.0306 (19)	0.030 (2)	0.038 (2)	0.0012 (17)	−0.0041 (17)	0.0007 (19)
N4	0.034 (2)	0.039 (2)	0.035 (2)	−0.0104 (19)	0.0021 (18)	−0.004 (2)
N5	0.0307 (18)	0.051 (3)	0.027 (2)	0.0190 (18)	0.0085 (16)	0.0074 (19)
N6	0.0251 (17)	0.036 (2)	0.028 (2)	0.0100 (16)	0.0072 (16)	0.0002 (17)
N7	0.024 (2)	0.122 (5)	0.032 (3)	0.016 (2)	−0.0065 (18)	0.012 (3)
N8	0.0289 (19)	0.058 (3)	0.036 (2)	0.0129 (19)	−0.0108 (18)	0.008 (2)
N9	0.0220 (18)	0.053 (3)	0.027 (2)	0.0054 (17)	−0.0037 (16)	0.0090 (19)
N10	0.0278 (18)	0.051 (3)	0.034 (2)	0.0080 (18)	−0.0099 (18)	−0.001 (2)
N11	0.0280 (19)	0.046 (3)	0.031 (2)	0.0125 (17)	0.0067 (16)	0.0009 (19)
N12	0.0301 (18)	0.052 (3)	0.025 (2)	0.0174 (18)	0.0068 (16)	−0.0013 (19)
N13	0.0265 (18)	0.037 (2)	0.050 (3)	0.0064 (17)	0.0151 (18)	0.015 (2)
N14	0.0285 (19)	0.049 (3)	0.036 (3)	−0.0062 (18)	0.0114 (18)	0.007 (2)
N15	0.039 (2)	0.035 (2)	0.032 (2)	0.0087 (18)	−0.0103 (18)	−0.006 (2)
N16	0.036 (2)	0.036 (2)	0.036 (2)	−0.0010 (18)	−0.0020 (18)	−0.003 (2)
C1	0.035 (2)	0.050 (3)	0.033 (3)	0.002 (2)	−0.006 (2)	0.010 (2)
C2	0.040 (3)	0.052 (3)	0.043 (3)	0.015 (2)	−0.016 (2)	0.009 (3)
C3	0.052 (3)	0.073 (5)	0.062 (4)	0.026 (3)	−0.010 (3)	0.009 (4)
C4	0.092 (5)	0.104 (7)	0.059 (5)	0.065 (5)	−0.023 (4)	−0.013 (5)
C5	0.100 (6)	0.056 (5)	0.099 (7)	0.032 (5)	−0.057 (5)	−0.006 (5)
C6	0.084 (5)	0.049 (4)	0.117 (7)	0.017 (4)	−0.034 (5)	0.023 (5)
C7	0.057 (3)	0.047 (4)	0.073 (5)	0.003 (3)	−0.019 (3)	0.024 (3)
C8	0.043 (2)	0.034 (3)	0.023 (2)	0.006 (2)	0.007 (2)	0.003 (2)
C9	0.028 (2)	0.051 (3)	0.041 (3)	0.002 (2)	0.004 (2)	0.019 (3)
C10	0.047 (3)	0.053 (3)	0.041 (3)	0.008 (3)	−0.005 (2)	0.020 (3)
C11	0.044 (3)	0.065 (4)	0.049 (4)	0.020 (3)	−0.011 (3)	0.005 (3)
C12	0.026 (2)	0.099 (5)	0.060 (4)	0.009 (3)	0.005 (3)	0.012 (4)
C13	0.033 (3)	0.082 (4)	0.040 (3)	0.002 (3)	0.015 (2)	0.016 (3)
C14	0.029 (2)	0.032 (2)	0.026 (2)	0.0082 (19)	0.0027 (18)	0.002 (2)
C15	0.055 (3)	0.039 (3)	0.044 (3)	0.014 (2)	0.018 (3)	0.014 (3)
C16	0.043 (3)	0.038 (3)	0.043 (3)	0.018 (2)	0.011 (2)	0.013 (2)
C17	0.048 (3)	0.078 (5)	0.065 (4)	0.026 (3)	0.014 (3)	0.029 (4)
C18	0.090 (5)	0.125 (8)	0.107 (7)	0.076 (5)	0.056 (5)	0.067 (7)
C19	0.157 (9)	0.091 (6)	0.081 (7)	0.087 (7)	0.063 (6)	0.029 (5)
C20	0.154 (8)	0.073 (5)	0.044 (4)	0.049 (6)	0.003 (5)	−0.001 (4)
C21	0.072 (4)	0.050 (4)	0.061 (4)	0.029 (3)	−0.005 (3)	0.001 (3)
C22	0.052 (3)	0.032 (3)	0.030 (3)	0.003 (2)	0.016 (2)	0.008 (2)
C23	0.055 (3)	0.043 (3)	0.028 (3)	0.010 (2)	0.011 (2)	0.009 (2)
C24	0.065 (3)	0.039 (3)	0.040 (3)	0.009 (3)	0.019 (3)	0.010 (3)
C25	0.109 (5)	0.045 (4)	0.039 (4)	0.016 (4)	0.014 (4)	0.016 (3)
C26	0.144 (7)	0.057 (5)	0.056 (5)	0.045 (5)	0.000 (5)	0.021 (4)
C27	0.108 (6)	0.089 (6)	0.070 (5)	0.039 (5)	−0.022 (4)	0.027 (5)
C28	0.075 (4)	0.062 (4)	0.048 (4)	0.005 (3)	−0.013 (3)	0.020 (3)
C29	0.030 (2)	0.033 (3)	0.037 (3)	0.0011 (19)	0.0042 (19)	0.001 (2)
C30	0.046 (3)	0.037 (3)	0.040 (3)	0.013 (2)	0.003 (2)	0.017 (2)
C31	0.040 (3)	0.073 (4)	0.043 (3)	0.010 (3)	0.007 (2)	0.020 (3)
C32	0.059 (4)	0.097 (5)	0.035 (3)	0.024 (4)	−0.003 (3)	0.007 (3)
C33	0.056 (3)	0.072 (4)	0.056 (4)	0.017 (3)	−0.014 (3)	0.014 (4)
C34	0.040 (3)	0.068 (4)	0.070 (5)	0.017 (3)	0.002 (3)	0.023 (4)
C35	0.052 (3)	0.060 (4)	0.044 (3)	0.021 (3)	0.009 (3)	0.017 (3)
C36	0.051 (3)	0.034 (3)	0.039 (3)	0.005 (2)	0.001 (2)	0.011 (2)
C37	0.025 (2)	0.033 (2)	0.026 (2)	0.0057 (18)	0.0033 (17)	0.002 (2)
C38	0.023 (2)	0.045 (3)	0.032 (3)	0.0093 (19)	0.0037 (18)	0.005 (2)
C39	0.032 (2)	0.038 (3)	0.044 (3)	0.000 (2)	0.009 (2)	0.002 (2)
C40	0.040 (3)	0.050 (3)	0.038 (3)	0.013 (2)	0.014 (2)	0.016 (3)
C41	0.044 (3)	0.060 (4)	0.027 (3)	0.013 (3)	0.008 (2)	0.008 (3)
C42	0.035 (2)	0.048 (3)	0.034 (3)	−0.001 (2)	0.001 (2)	−0.001 (2)
C43	0.043 (3)	0.037 (3)	0.032 (3)	0.011 (2)	0.008 (2)	0.010 (2)
C44	0.035 (2)	0.032 (2)	0.028 (3)	0.0156 (19)	0.0008 (19)	0.008 (2)
C45	0.039 (3)	0.082 (5)	0.045 (4)	0.004 (3)	−0.006 (2)	0.014 (3)
C46	0.069 (4)	0.087 (6)	0.072 (5)	−0.005 (4)	−0.038 (4)	0.020 (4)
C47	0.139 (7)	0.045 (4)	0.039 (4)	0.026 (4)	−0.023 (4)	0.005 (3)
C48	0.099 (5)	0.091 (6)	0.037 (4)	0.034 (5)	0.016 (4)	0.011 (4)
C49	0.051 (3)	0.087 (5)	0.034 (3)	0.019 (3)	0.014 (2)	0.016 (3)
C50	0.045 (2)	0.026 (2)	0.025 (3)	0.009 (2)	0.005 (2)	−0.002 (2)
C51	0.034 (2)	0.022 (2)	0.033 (3)	−0.0007 (18)	0.0056 (19)	0.003 (2)
C52	0.040 (3)	0.034 (3)	0.039 (3)	−0.003 (2)	−0.006 (2)	0.007 (2)
C53	0.033 (2)	0.039 (3)	0.056 (4)	0.000 (2)	0.003 (2)	0.013 (3)
C54	0.046 (3)	0.059 (4)	0.046 (3)	0.011 (3)	0.019 (2)	0.026 (3)
C55	0.046 (3)	0.054 (3)	0.040 (3)	0.008 (3)	0.005 (2)	0.022 (3)
C56	0.034 (2)	0.042 (3)	0.037 (3)	0.005 (2)	−0.002 (2)	0.013 (2)
C57	0.027 (2)	0.035 (3)	0.030 (3)	0.0058 (19)	−0.0003 (18)	0.008 (2)
C58	0.0204 (19)	0.038 (3)	0.031 (3)	0.0047 (18)	−0.0054 (17)	0.005 (2)
C59	0.029 (2)	0.052 (3)	0.051 (3)	0.007 (2)	0.003 (2)	0.014 (3)
C60	0.052 (3)	0.058 (4)	0.072 (5)	−0.009 (3)	0.013 (3)	0.028 (4)
C61	0.088 (5)	0.034 (3)	0.097 (6)	−0.002 (3)	0.009 (4)	0.010 (4)
C62	0.081 (4)	0.035 (3)	0.086 (6)	0.012 (3)	0.019 (4)	−0.003 (3)
C63	0.044 (3)	0.034 (3)	0.047 (3)	0.001 (2)	0.008 (2)	−0.007 (2)
C64A	0.060 (10)	0.111 (15)	0.037 (6)	0.051 (10)	−0.006 (5)	0.005 (6)
C64B	0.018 (9)	0.048 (15)	0.07 (2)	0.003 (8)	−0.013 (9)	−0.022 (13)
C65	0.048 (3)	0.135 (7)	0.034 (3)	0.059 (4)	−0.014 (3)	−0.010 (4)
C66	0.082 (5)	0.174 (10)	0.073 (6)	0.080 (6)	0.027 (4)	0.070 (7)
C67	0.082 (5)	0.128 (9)	0.140 (10)	0.055 (6)	0.018 (6)	0.089 (8)
C68	0.067 (4)	0.076 (5)	0.120 (8)	0.044 (4)	−0.047 (5)	−0.013 (5)
C69	0.052 (3)	0.145 (8)	0.039 (4)	0.038 (4)	−0.002 (3)	−0.025 (4)
C70	0.030 (3)	0.102 (6)	0.066 (5)	0.011 (3)	0.005 (3)	−0.012 (4)
C71	0.033 (2)	0.054 (3)	0.038 (3)	0.022 (2)	−0.004 (2)	−0.006 (3)
C72	0.0202 (19)	0.060 (3)	0.030 (3)	0.021 (2)	−0.0011 (18)	−0.001 (2)
C73	0.046 (3)	0.056 (3)	0.031 (3)	0.023 (3)	−0.009 (2)	0.001 (3)
C74	0.051 (3)	0.051 (3)	0.050 (4)	0.022 (3)	−0.013 (3)	0.000 (3)
C75	0.051 (3)	0.073 (4)	0.040 (3)	0.028 (3)	0.013 (3)	0.017 (3)
C76	0.044 (3)	0.095 (5)	0.031 (3)	0.025 (3)	0.007 (2)	0.012 (3)
C77	0.035 (3)	0.085 (5)	0.029 (3)	0.011 (3)	−0.002 (2)	−0.001 (3)
C78A	0.062 (6)	0.054 (9)	0.077 (11)	−0.006 (6)	−0.031 (7)	0.030 (7)
C79A	0.056 (10)	0.071 (11)	0.038 (8)	0.012 (7)	−0.025 (7)	0.007 (7)
C80A	0.046 (3)	0.081 (5)	0.074 (5)	0.019 (3)	0.001 (3)	0.023 (4)
C81A	0.046 (3)	0.081 (5)	0.074 (5)	0.019 (3)	0.001 (3)	0.023 (4)
C82A	0.046 (3)	0.081 (5)	0.074 (5)	0.019 (3)	0.001 (3)	0.023 (4)
C83A	0.046 (3)	0.081 (5)	0.074 (5)	0.019 (3)	0.001 (3)	0.023 (4)
C84A	0.046 (3)	0.081 (5)	0.074 (5)	0.019 (3)	0.001 (3)	0.023 (4)
C78B	0.062 (6)	0.054 (9)	0.077 (11)	−0.006 (6)	−0.031 (7)	0.030 (7)
C79B	0.029 (6)	0.034 (6)	0.035 (7)	0.001 (5)	0.016 (5)	0.019 (6)
C80B	0.070 (4)	0.042 (4)	0.135 (8)	−0.001 (3)	−0.007 (5)	0.005 (4)
C81B	0.070 (4)	0.042 (4)	0.135 (8)	−0.001 (3)	−0.007 (5)	0.005 (4)
C82B	0.070 (4)	0.042 (4)	0.135 (8)	−0.001 (3)	−0.007 (5)	0.005 (4)
C83B	0.070 (4)	0.042 (4)	0.135 (8)	−0.001 (3)	−0.007 (5)	0.005 (4)
C84B	0.070 (4)	0.042 (4)	0.135 (8)	−0.001 (3)	−0.007 (5)	0.005 (4)
C85	0.031 (2)	0.047 (3)	0.024 (3)	0.003 (2)	−0.0031 (18)	0.006 (2)
C86	0.0230 (19)	0.044 (3)	0.023 (2)	0.0053 (19)	0.0057 (17)	0.010 (2)
C87	0.0210 (19)	0.046 (3)	0.031 (3)	0.0124 (19)	0.0020 (17)	0.013 (2)
C88	0.036 (2)	0.035 (3)	0.039 (3)	0.011 (2)	0.009 (2)	0.010 (2)
C89	0.031 (2)	0.040 (3)	0.031 (3)	0.002 (2)	0.0043 (19)	0.011 (2)
C90	0.026 (2)	0.044 (3)	0.036 (3)	0.007 (2)	0.0020 (19)	0.012 (2)
C91	0.027 (2)	0.040 (3)	0.037 (3)	0.0114 (19)	0.0093 (19)	0.016 (2)
C92	0.029 (2)	0.037 (3)	0.051 (3)	0.002 (2)	0.004 (2)	0.016 (2)
C93	0.028 (2)	0.033 (3)	0.067 (4)	0.003 (2)	0.004 (2)	0.014 (3)
C94	0.039 (3)	0.065 (4)	0.102 (6)	0.014 (3)	−0.015 (3)	0.020 (4)
C95	0.049 (4)	0.073 (5)	0.148 (9)	0.020 (4)	0.005 (5)	0.028 (6)
C96	0.039 (3)	0.062 (5)	0.174 (11)	0.018 (3)	0.031 (5)	0.028 (6)
C97	0.094 (6)	0.060 (5)	0.107 (7)	0.016 (4)	0.043 (5)	0.007 (5)
C98	0.065 (4)	0.052 (4)	0.070 (5)	0.015 (3)	0.012 (3)	0.005 (3)
C99	0.026 (2)	0.048 (3)	0.029 (3)	0.010 (2)	0.0020 (18)	0.006 (2)
C100	0.025 (2)	0.048 (3)	0.024 (2)	0.012 (2)	0.0063 (17)	0.005 (2)
C101	0.039 (3)	0.061 (4)	0.042 (3)	0.025 (3)	0.006 (2)	0.016 (3)
C102	0.058 (3)	0.080 (5)	0.060 (4)	0.042 (3)	0.019 (3)	0.036 (4)
C103	0.069 (4)	0.047 (4)	0.084 (5)	0.027 (3)	0.046 (4)	0.025 (4)
C104	0.047 (3)	0.040 (3)	0.063 (4)	0.009 (3)	0.019 (3)	0.003 (3)
C105	0.035 (2)	0.042 (3)	0.035 (3)	0.008 (2)	0.003 (2)	0.002 (2)
C106	0.024 (2)	0.051 (3)	0.031 (3)	0.009 (2)	0.0003 (18)	0.007 (2)
C107	0.024 (2)	0.038 (3)	0.034 (3)	0.0092 (19)	−0.0043 (18)	0.006 (2)
C108	0.035 (2)	0.046 (3)	0.051 (3)	0.014 (2)	0.015 (2)	0.004 (3)
C109	0.048 (3)	0.036 (3)	0.070 (4)	0.007 (2)	0.013 (3)	0.011 (3)
C110	0.043 (3)	0.053 (3)	0.048 (3)	0.012 (2)	0.009 (2)	0.021 (3)
C111	0.066 (3)	0.053 (4)	0.028 (3)	0.016 (3)	0.002 (2)	0.005 (3)
C112	0.059 (3)	0.040 (3)	0.033 (3)	0.017 (2)	−0.003 (2)	0.004 (2)
C113	0.028 (2)	0.037 (3)	0.031 (3)	0.0042 (19)	−0.0009 (18)	0.006 (2)
C114	0.028 (2)	0.033 (3)	0.041 (3)	0.0079 (19)	0.0023 (19)	0.014 (2)
C115	0.048 (3)	0.059 (4)	0.041 (3)	0.018 (3)	−0.005 (2)	0.002 (3)
C116	0.073 (4)	0.069 (5)	0.057 (4)	0.006 (4)	−0.020 (3)	0.009 (4)
C117	0.054 (4)	0.093 (6)	0.114 (8)	−0.025 (4)	−0.036 (4)	0.059 (6)
C118	0.027 (3)	0.135 (8)	0.120 (7)	0.028 (4)	0.017 (4)	0.080 (7)
C119	0.044 (3)	0.097 (5)	0.067 (4)	0.040 (3)	0.025 (3)	0.047 (4)
C120	0.022 (2)	0.043 (3)	0.053 (3)	0.001 (2)	0.007 (2)	−0.001 (3)
C121	0.0181 (19)	0.040 (3)	0.049 (3)	0.0010 (19)	−0.0028 (19)	0.004 (2)
C122	0.033 (2)	0.052 (3)	0.050 (4)	0.005 (2)	−0.012 (2)	−0.002 (3)
C123	0.041 (3)	0.069 (5)	0.059 (4)	0.012 (3)	−0.013 (3)	−0.021 (4)
C124	0.034 (3)	0.037 (3)	0.112 (7)	0.004 (2)	−0.007 (3)	−0.012 (4)
C125	0.034 (3)	0.044 (4)	0.100 (6)	0.004 (2)	0.004 (3)	0.028 (4)
C126	0.028 (2)	0.044 (3)	0.059 (4)	0.001 (2)	0.007 (2)	0.015 (3)
C127	0.029 (2)	0.031 (2)	0.034 (3)	0.0050 (19)	0.0031 (19)	−0.005 (2)
C128	0.031 (2)	0.039 (3)	0.032 (3)	0.006 (2)	0.0091 (19)	0.002 (2)
C129	0.075 (4)	0.049 (4)	0.043 (4)	0.004 (3)	−0.001 (3)	0.017 (3)
C130	0.094 (5)	0.064 (4)	0.045 (4)	0.029 (4)	0.009 (3)	0.027 (3)
C131	0.056 (3)	0.091 (5)	0.051 (4)	0.027 (4)	0.010 (3)	0.032 (4)
C132	0.045 (3)	0.076 (5)	0.072 (5)	−0.001 (3)	−0.013 (3)	0.024 (4)
C133	0.041 (3)	0.053 (4)	0.055 (4)	0.002 (3)	−0.007 (3)	0.019 (3)
C134	0.035 (2)	0.066 (4)	0.037 (3)	0.010 (2)	−0.001 (2)	0.014 (3)
C135	0.033 (2)	0.061 (4)	0.048 (3)	0.021 (2)	0.002 (2)	0.021 (3)
C136	0.067 (4)	0.068 (5)	0.124 (7)	0.035 (4)	0.037 (4)	0.050 (5)
C137	0.091 (6)	0.065 (6)	0.199 (12)	0.034 (5)	0.032 (7)	0.050 (7)
C138	0.101 (6)	0.073 (6)	0.152 (10)	0.052 (5)	0.000 (6)	0.012 (6)
C139	0.082 (5)	0.096 (6)	0.078 (5)	0.071 (5)	−0.002 (4)	0.000 (5)
C140	0.041 (3)	0.082 (5)	0.055 (4)	0.031 (3)	0.004 (3)	0.018 (3)
C141	0.035 (2)	0.038 (3)	0.051 (3)	0.005 (2)	0.013 (2)	0.013 (3)
C142	0.042 (3)	0.035 (3)	0.042 (3)	0.018 (2)	0.014 (2)	0.013 (2)
C143	0.083 (4)	0.054 (4)	0.046 (4)	0.026 (3)	0.009 (3)	0.004 (3)
C144	0.115 (6)	0.077 (5)	0.045 (4)	0.046 (5)	0.037 (4)	0.011 (4)
C145	0.066 (4)	0.087 (5)	0.080 (6)	0.045 (4)	0.030 (4)	0.030 (4)
C146	0.050 (3)	0.092 (5)	0.079 (5)	0.038 (3)	0.016 (3)	0.041 (4)
C147	0.046 (3)	0.065 (4)	0.049 (4)	0.021 (3)	0.008 (2)	0.025 (3)
C148	0.040 (2)	0.038 (3)	0.041 (3)	0.007 (2)	0.002 (2)	0.001 (2)
C149	0.046 (3)	0.038 (3)	0.028 (3)	0.010 (2)	0.003 (2)	0.002 (2)
C151	0.066 (4)	0.040 (3)	0.066 (4)	0.006 (3)	−0.001 (3)	0.003 (3)
C152	0.131 (7)	0.033 (3)	0.075 (5)	0.017 (4)	−0.001 (5)	0.007 (3)
C153	0.142 (7)	0.074 (5)	0.055 (5)	0.070 (5)	−0.003 (5)	0.016 (4)
C154	0.090 (5)	0.078 (5)	0.065 (5)	0.054 (4)	−0.007 (4)	0.013 (4)
C155	0.057 (3)	0.062 (4)	0.053 (4)	0.019 (3)	−0.012 (3)	0.006 (3)
C156	0.031 (2)	0.057 (3)	0.041 (3)	0.021 (2)	0.001 (2)	0.000 (3)
C157	0.028 (2)	0.046 (3)	0.045 (3)	0.012 (2)	0.006 (2)	0.002 (3)
C158	0.039 (3)	0.054 (4)	0.081 (5)	−0.005 (3)	0.001 (3)	0.002 (4)
C159	0.061 (4)	0.063 (5)	0.130 (8)	−0.011 (4)	0.012 (5)	0.046 (5)
C160	0.071 (5)	0.110 (8)	0.099 (7)	0.015 (5)	0.024 (5)	0.057 (6)
C161	0.093 (5)	0.101 (6)	0.049 (4)	0.041 (5)	0.019 (4)	0.013 (4)
C162	0.067 (4)	0.053 (4)	0.054 (4)	0.024 (3)	0.013 (3)	0.005 (3)
C163	0.046 (3)	0.034 (3)	0.056 (4)	0.018 (2)	−0.006 (3)	−0.004 (3)
C164	0.038 (2)	0.028 (3)	0.062 (4)	0.012 (2)	−0.011 (2)	0.008 (3)
C165	0.042 (3)	0.060 (4)	0.063 (4)	0.011 (3)	−0.011 (3)	0.018 (3)
C166	0.067 (4)	0.102 (6)	0.053 (4)	0.028 (4)	−0.005 (3)	0.016 (4)
C167	0.085 (5)	0.065 (5)	0.069 (5)	0.029 (4)	−0.037 (4)	0.004 (4)
C168	0.049 (3)	0.054 (4)	0.093 (6)	0.004 (3)	−0.032 (4)	0.017 (4)
C169	0.035 (3)	0.059 (4)	0.082 (5)	0.006 (3)	−0.013 (3)	0.022 (4)
C201	0.0233 (19)	0.025 (2)	0.032 (3)	0.0003 (17)	−0.0028 (18)	−0.001 (2)
C202	0.025 (2)	0.034 (3)	0.028 (3)	0.0041 (18)	0.0015 (18)	0.005 (2)
C203	0.032 (2)	0.028 (2)	0.023 (2)	0.0074 (19)	0.0065 (18)	0.0053 (19)
C204	0.024 (2)	0.043 (3)	0.028 (3)	0.003 (2)	0.0089 (18)	0.006 (2)
C205	0.0240 (19)	0.033 (2)	0.027 (2)	0.0085 (18)	−0.0014 (18)	0.005 (2)
C206	0.0175 (18)	0.031 (2)	0.024 (2)	0.0040 (17)	−0.0038 (16)	0.0068 (19)
C207	0.028 (2)	0.060 (3)	0.026 (3)	0.014 (2)	0.0083 (19)	0.011 (2)
C208	0.031 (2)	0.044 (3)	0.022 (2)	0.012 (2)	0.0059 (19)	0.004 (2)
C209	0.022 (2)	0.043 (3)	0.017 (2)	0.0089 (18)	0.0070 (17)	0.005 (2)
C210	0.0204 (19)	0.035 (3)	0.031 (3)	0.0026 (18)	−0.0012 (18)	0.002 (2)
C211	0.0228 (19)	0.033 (2)	0.019 (2)	0.0053 (17)	−0.0046 (17)	−0.0023 (19)
C212	0.0164 (18)	0.043 (3)	0.030 (3)	0.0109 (18)	−0.0031 (17)	0.006 (2)
C213	0.026 (2)	0.026 (2)	0.027 (2)	0.0037 (17)	−0.0017 (18)	0.0007 (19)
C214	0.0216 (19)	0.030 (2)	0.031 (3)	−0.0051 (18)	−0.0048 (18)	−0.002 (2)
C215	0.026 (2)	0.024 (2)	0.029 (3)	0.0089 (17)	0.0067 (18)	0.003 (2)
C216	0.0240 (19)	0.031 (3)	0.028 (2)	0.0017 (18)	0.0026 (18)	0.004 (2)

### Poly[hexabenzyltetra-µ-cyanato-nickel(II)ditin(IV)] Geometric parameters (Å, º)

**Table:**

Sn1—C8	2.140 (5)	C69—C70	1.3900
Sn1—C15	2.145 (5)	C69—H69	0.9500
Sn1—C1	2.152 (5)	C70—H70	0.9500
Sn1—N1	2.373 (4)	C71—C72	1.480 (8)
Sn1—N2	2.274 (4)	C71—H71A	0.9900
Sn2—C29	2.133 (5)	C71—H71B	0.9900
Sn2—C22	2.133 (5)	C72—C73	1.378 (7)
Sn2—C36	2.142 (5)	C72—C77	1.388 (7)
Sn2—N3	2.273 (4)	C73—C74	1.384 (8)
Sn2—N4	2.359 (4)	C73—H73	0.9500
Sn3—C50	2.137 (4)	C74—C75	1.379 (8)
Sn3—C43	2.138 (5)	C74—H74	0.9500
Sn3—C57	2.139 (4)	C75—C76	1.371 (9)
Sn3—N5	2.321 (4)	C75—H75	0.9500
Sn3—N6	2.330 (3)	C76—C77	1.366 (9)
Sn4—C64B	1.98 (2)	C76—H76	0.9500
Sn4—C78A	2.076 (15)	C77—H77	0.9500
Sn4—C71	2.130 (5)	C78A—C79A	1.490 (12)
Sn4—C64A	2.192 (16)	C78A—H78A	0.9900
Sn4—C78B	2.266 (16)	C78A—H78B	0.9900
Sn4—N7	2.311 (4)	C79A—C80A	1.3900
Sn4—N8	2.342 (4)	C79A—C84A	1.3900
Sn5—C92	2.126 (5)	C80A—C81A	1.3900
Sn5—C99	2.138 (5)	C80A—H80A	0.9500
Sn5—C85	2.142 (4)	C81A—C82A	1.3900
Sn5—N9	2.303 (4)	C81A—H81A	0.9500
Sn5—N10	2.335 (4)	C82A—C83A	1.3900
Sn6—C113	2.143 (5)	C82A—H82A	0.9500
Sn6—C106	2.155 (5)	C83A—C84A	1.3900
Sn6—C120	2.162 (4)	C83A—H83A	0.9500
Sn6—N11	2.340 (4)	C84A—H84A	0.9500
Sn6—N12	2.296 (4)	C78B—C79B	1.493 (13)
Sn7—C127	2.142 (5)	C78B—H78C	0.9900
Sn7—C141	2.144 (5)	C78B—H78D	0.9900
Sn7—C134	2.150 (5)	C79B—C80B	1.3900
Sn7—N13	2.260 (3)	C79B—C84B	1.3900
Sn7—N14	2.394 (4)	C80B—C81B	1.3900
Sn8—C148	2.140 (5)	C80B—H80B	0.9500
Sn8—C163	2.153 (5)	C81B—C82B	1.3900
Sn8—C156	2.154 (5)	C81B—H81B	0.9500
Sn8—N15	2.244 (4)	C82B—C83B	1.3900
Sn8—N16	2.387 (4)	C82B—H82B	0.9500
Ni1—C203	1.848 (4)	C83B—C84B	1.3900
Ni1—C202	1.852 (5)	C83B—H83B	0.9500
Ni1—C205	1.854 (4)	C84B—H84B	0.9500
Ni1—C207	1.863 (5)	C85—C86	1.507 (6)
Ni2—C209	1.854 (4)	C85—H85A	0.9900
Ni2—C212	1.856 (5)	C85—H85B	0.9900
Ni2—C208^i^	1.860 (5)	C86—C87	1.358 (7)
Ni2—C206	1.862 (4)	C86—C91	1.392 (6)
Ni3—C215	1.841 (5)	C87—C88	1.387 (7)
Ni3—C211^ii^	1.846 (4)	C87—H87	0.9500
Ni3—C210	1.856 (5)	C88—C89	1.386 (7)
Ni3—C213	1.858 (4)	C88—H88	0.9500
Ni4—C214^iii^	1.848 (5)	C89—C90	1.367 (7)
Ni4—C216^iv^	1.855 (5)	C89—H89	0.9500
Ni4—C201	1.855 (5)	C90—C91	1.379 (7)
Ni4—C204^v^	1.857 (5)	C90—H90	0.9500
N1—C201	1.148 (6)	C91—H91	0.9500
N2—C202	1.153 (6)	C92—C93	1.493 (7)
N3—C203	1.149 (5)	C92—H92A	0.9900
N4—C204	1.144 (6)	C92—H92B	0.9900
N5—C205	1.152 (6)	C93—C98	1.357 (9)
N6—C206	1.132 (6)	C93—C94	1.397 (8)
N7—C207	1.153 (6)	C94—C95	1.384 (10)
N8—C208	1.160 (6)	C94—H94	0.9500
N9—C209	1.146 (5)	C95—C96	1.318 (13)
N10—C210	1.133 (6)	C95—H95	0.9500
N11—C211	1.145 (5)	C96—C97	1.367 (13)
N12—C212	1.140 (6)	C96—H96	0.9500
N13—C213	1.145 (5)	C97—C98	1.413 (10)
N14—C214	1.158 (6)	C97—H97	0.9500
N15—C215	1.148 (6)	C98—H98	0.9500
N16—C216	1.142 (6)	C99—C100	1.466 (7)
C1—C2	1.460 (8)	C99—H99A	0.9900
C1—H1A	0.9900	C99—H99B	0.9900
C1—H1B	0.9900	C100—C101	1.399 (7)
C2—C3	1.377 (9)	C100—C105	1.399 (7)
C2—C7	1.383 (8)	C101—C102	1.366 (9)
C3—C4	1.406 (10)	C101—H10A	0.9500
C3—H3	0.9500	C102—C103	1.380 (10)
C4—C5	1.384 (12)	C102—H10B	0.9500
C4—H4	0.9500	C103—C104	1.362 (9)
C5—C6	1.318 (12)	C103—H10C	0.9500
C5—H5	0.9500	C104—C105	1.363 (8)
C6—C7	1.357 (10)	C104—H10D	0.9500
C6—H6	0.9500	C105—H10E	0.9500
C7—H7	0.9500	C106—C107	1.500 (7)
C8—C14	1.502 (7)	C106—H10F	0.9900
C8—H8A	0.9900	C106—H10G	0.9900
C8—H8B	0.9900	C107—C108	1.363 (7)
C9—C14	1.377 (7)	C107—C112	1.372 (7)
C9—C10	1.379 (7)	C108—C109	1.386 (8)
C9—H9	0.9500	C108—H10H	0.9500
C10—C11	1.376 (7)	C109—C110	1.384 (8)
C10—H10	0.9500	C109—H10I	0.9500
C11—C12	1.361 (9)	C110—C111	1.349 (8)
C11—H11	0.9500	C110—H11A	0.9500
C12—C13	1.383 (9)	C111—C112	1.405 (8)
C12—H12	0.9500	C111—H11B	0.9500
C13—C14	1.390 (6)	C112—H11C	0.9500
C13—H13	0.9500	C113—C114	1.489 (6)
C15—C16	1.479 (7)	C113—H11D	0.9900
C15—H15A	0.9900	C113—H11E	0.9900
C15—H15B	0.9900	C114—C115	1.375 (8)
C16—C17	1.379 (7)	C114—C119	1.378 (7)
C16—C21	1.385 (8)	C115—C116	1.383 (8)
C17—C18	1.357 (10)	C115—H11F	0.9500
C17—H17	0.9500	C116—C117	1.356 (11)
C18—C19	1.337 (13)	C116—H11G	0.9500
C18—H18	0.9500	C117—C118	1.348 (12)
C19—C20	1.352 (12)	C117—H11H	0.9500
C19—H19	0.9500	C118—C119	1.428 (10)
C20—C21	1.390 (10)	C118—H11I	0.9500
C20—H20	0.9500	C119—H11J	0.9500
C21—H21	0.9500	C120—C121	1.475 (7)
C22—C23	1.491 (8)	C120—H12A	0.9900
C22—H22A	0.9900	C120—H12B	0.9900
C22—H22B	0.9900	C121—C122	1.386 (8)
C23—C24	1.377 (7)	C121—C126	1.391 (7)
C23—C28	1.388 (8)	C122—C123	1.377 (9)
C24—C25	1.384 (9)	C122—H12C	0.9500
C24—H24	0.9500	C123—C124	1.349 (10)
C25—C26	1.379 (11)	C123—H12D	0.9500
C25—H25	0.9500	C124—C125	1.381 (10)
C26—C27	1.342 (11)	C124—H12E	0.9500
C26—H26	0.9500	C125—C126	1.376 (9)
C27—C28	1.378 (10)	C125—H12F	0.9500
C27—H27	0.9500	C126—H12G	0.9500
C28—H28	0.9500	C127—C128	1.497 (7)
C29—C37	1.497 (6)	C127—H12H	0.9900
C29—H29A	0.9900	C127—H12I	0.9900
C29—H29B	0.9900	C128—C133	1.377 (7)
C30—C31	1.373 (8)	C128—C129	1.378 (8)
C30—C35	1.385 (7)	C129—C130	1.387 (9)
C30—C36	1.488 (7)	C129—H12J	0.9500
C31—C32	1.366 (8)	C130—C131	1.377 (9)
C31—H31	0.9500	C130—H13A	0.9500
C32—C33	1.377 (8)	C131—C132	1.354 (9)
C32—H32	0.9500	C131—H13B	0.9500
C33—C34	1.356 (9)	C132—C133	1.382 (8)
C33—H33	0.9500	C132—H13C	0.9500
C34—C35	1.383 (8)	C133—H13D	0.9500
C34—H34	0.9500	C134—C135	1.461 (8)
C35—H35	0.9500	C134—H13E	0.9900
C36—H36A	0.9900	C134—H13F	0.9900
C36—H36B	0.9900	C135—C136	1.385 (8)
C37—C42	1.384 (7)	C135—C140	1.390 (8)
C37—C38	1.390 (6)	C136—C137	1.391 (12)
C38—C39	1.374 (7)	C136—H13G	0.9500
C38—H38	0.9500	C137—C138	1.341 (14)
C39—C40	1.374 (8)	C137—H13H	0.9500
C39—H39	0.9500	C138—C139	1.365 (12)
C40—C41	1.383 (7)	C138—H13I	0.9500
C40—H40	0.9500	C139—C140	1.365 (10)
C41—C42	1.372 (7)	C139—H13J	0.9500
C41—H41	0.9500	C140—H14A	0.9500
C42—H42	0.9500	C141—C142	1.495 (6)
C43—C44	1.497 (6)	C141—H14B	0.9900
C43—H43A	0.9900	C141—H14C	0.9900
C43—H43B	0.9900	C142—C143	1.369 (8)
C44—C45	1.3900	C142—C147	1.404 (7)
C44—C49	1.3900	C143—C144	1.433 (9)
C45—C46	1.3900	C143—H14D	0.9500
C45—H45	0.9500	C144—C145	1.356 (10)
C46—C47	1.3900	C144—H14E	0.9500
C46—H46	0.9500	C145—C146	1.336 (10)
C47—C48	1.3900	C145—H14F	0.9500
C47—H47	0.9500	C146—C147	1.359 (8)
C48—C49	1.3900	C146—H14G	0.9500
C48—H48	0.9500	C147—H14H	0.9500
C49—H49	0.9500	C148—C149	1.478 (7)
C50—C51	1.503 (6)	C148—H14I	0.9900
C50—H50A	0.9900	C148—H14J	0.9900
C50—H50B	0.9900	C149—C151	1.375 (8)
C51—C56	1.377 (7)	C149—C155	1.381 (8)
C51—C52	1.383 (7)	C151—C152	1.391 (10)
C52—C53	1.392 (7)	C151—H15C	0.9500
C52—H52	0.9500	C152—C153	1.371 (11)
C53—C54	1.370 (8)	C152—H15D	0.9500
C53—H53	0.9500	C153—C154	1.349 (11)
C54—C55	1.376 (7)	C153—H15E	0.9500
C54—H54	0.9500	C154—C155	1.362 (9)
C55—C56	1.387 (7)	C154—H15F	0.9500
C55—H55	0.9500	C155—H15G	0.9500
C56—H56	0.9500	C156—C157	1.479 (8)
C57—C58	1.484 (6)	C156—H15H	0.9900
C57—H57A	0.9900	C156—H15I	0.9900
C57—H57B	0.9900	C157—C158	1.373 (8)
C58—C63	1.376 (7)	C157—C162	1.385 (8)
C58—C59	1.388 (7)	C158—C159	1.384 (11)
C59—C60	1.386 (8)	C158—H15K	0.9500
C59—H59	0.9500	C159—C160	1.351 (12)
C60—C61	1.362 (10)	C159—H15L	0.9500
C60—H60	0.9500	C160—C161	1.367 (12)
C61—C62	1.380 (10)	C160—H16A	0.9500
C61—H61	0.9500	C161—C162	1.365 (10)
C62—C63	1.382 (8)	C161—H16B	0.9500
C62—H62	0.9500	C162—H16C	0.9500
C63—H63	0.9500	C163—C164	1.486 (8)
C64A—C65	1.434 (16)	C163—H16D	0.9900
C64A—H64A	0.9900	C163—H16E	0.9900
C64A—H64B	0.9900	C164—C165	1.386 (8)
C64B—C65	1.69 (2)	C164—C169	1.394 (7)
C64B—H64C	0.9900	C165—C166	1.384 (9)
C64B—H64D	0.9900	C165—H16F	0.9500
C65—C66	1.3900	C166—C167	1.384 (10)
C65—C70	1.3900	C166—H16G	0.9500
C66—C67	1.3900	C167—C168	1.362 (10)
C66—H66	0.9500	C167—H16H	0.9500
C67—C68	1.3900	C168—C169	1.373 (10)
C67—H67	0.9500	C168—H16I	0.9500
C68—C69	1.3900	C169—H16J	0.9500
C68—H68	0.9500		
			
C203—Ni1—C202	89.32 (18)	C72—C71—H71A	107.8
C203—Ni1—C205	88.88 (18)	Sn4—C71—H71A	107.8
C202—Ni1—C205	172.7 (2)	C72—C71—H71B	107.8
C203—Ni1—C207	172.7 (2)	Sn4—C71—H71B	107.8
C202—Ni1—C207	91.63 (19)	H71A—C71—H71B	107.2
C205—Ni1—C207	91.05 (19)	C73—C72—C77	117.4 (5)
C209—Ni2—C212	88.69 (17)	C73—C72—C71	121.5 (5)
C209—Ni2—C208^i^	178.9 (2)	C77—C72—C71	121.1 (5)
C212—Ni2—C208^i^	91.85 (18)	C72—C73—C74	121.3 (5)
C209—Ni2—C206	88.55 (17)	C72—C73—H73	119.4
C212—Ni2—C206	177.2 (2)	C74—C73—H73	119.4
C208^i^—Ni2—C206	90.90 (18)	C75—C74—C73	120.1 (6)
C215—Ni3—C211^ii^	88.90 (18)	C75—C74—H74	120.0
C215—Ni3—C210	173.0 (2)	C73—C74—H74	120.0
C211^ii^—Ni3—C210	89.18 (18)	C76—C75—C74	119.0 (6)
C215—Ni3—C213	91.43 (18)	C76—C75—H75	120.5
C211^ii^—Ni3—C213	174.2 (2)	C74—C75—H75	120.5
C210—Ni3—C213	91.17 (19)	C77—C76—C75	120.7 (5)
C214^iii^—Ni4—C216^iv^	88.54 (19)	C77—C76—H76	119.6
C214^iii^—Ni4—C201	179.0 (2)	C75—C76—H76	119.6
C216^iv^—Ni4—C201	91.41 (19)	C76—C77—C72	121.4 (5)
C214^iii^—Ni4—C204^v^	91.53 (19)	C76—C77—H77	119.3
C216^iv^—Ni4—C204^v^	177.8 (2)	C72—C77—H77	119.3
C201—Ni4—C204^v^	88.55 (18)	C79A—C78A—Sn4	115.2 (10)
C8—Sn1—C15	122.2 (2)	C79A—C78A—H78A	108.5
C8—Sn1—C1	110.4 (2)	Sn4—C78A—H78A	108.5
C15—Sn1—C1	127.0 (2)	C79A—C78A—H78B	108.5
C8—Sn1—N2	94.13 (16)	Sn4—C78A—H78B	108.5
C15—Sn1—N2	93.41 (17)	H78A—C78A—H78B	107.5
C1—Sn1—N2	88.88 (18)	C80A—C79A—C84A	120.0
C8—Sn1—N1	88.50 (16)	C80A—C79A—C78A	123.8 (10)
C15—Sn1—N1	86.11 (17)	C84A—C79A—C78A	115.8 (10)
C1—Sn1—N1	89.17 (17)	C79A—C80A—C81A	120.0
N2—Sn1—N1	177.2 (2)	C79A—C80A—H80A	120.0
C29—Sn2—C22	121.0 (2)	C81A—C80A—H80A	120.0
C29—Sn2—C36	118.2 (2)	C80A—C81A—C82A	120.0
C22—Sn2—C36	120.4 (2)	C80A—C81A—H81A	120.0
C29—Sn2—N3	93.16 (16)	C82A—C81A—H81A	120.0
C22—Sn2—N3	88.24 (18)	C83A—C82A—C81A	120.0
C36—Sn2—N3	94.54 (17)	C83A—C82A—H82A	120.0
C29—Sn2—N4	88.22 (17)	C81A—C82A—H82A	120.0
C22—Sn2—N4	89.99 (17)	C82A—C83A—C84A	120.0
C36—Sn2—N4	85.89 (18)	C82A—C83A—H83A	120.0
N3—Sn2—N4	178.15 (14)	C84A—C83A—H83A	120.0
C50—Sn3—C43	127.12 (19)	C83A—C84A—C79A	120.0
C50—Sn3—C57	118.52 (19)	C83A—C84A—H84A	120.0
C43—Sn3—C57	114.36 (18)	C79A—C84A—H84A	120.0
C50—Sn3—N5	89.56 (16)	C79B—C78B—Sn4	114.8 (10)
C43—Sn3—N5	88.68 (17)	C79B—C78B—H78C	108.6
C57—Sn3—N5	92.85 (16)	Sn4—C78B—H78C	108.6
C50—Sn3—N6	91.73 (15)	C79B—C78B—H78D	108.6
C43—Sn3—N6	88.44 (17)	Sn4—C78B—H78D	108.6
C57—Sn3—N6	88.84 (16)	H78C—C78B—H78D	107.5
N5—Sn3—N6	177.07 (14)	C80B—C79B—C84B	120.0
C64B—Sn4—C71	140.3 (14)	C80B—C79B—C78B	125.6 (11)
C78A—Sn4—C71	127.0 (6)	C84B—C79B—C78B	114.3 (11)
C78A—Sn4—C64A	115.3 (9)	C79B—C80B—C81B	120.0
C71—Sn4—C64A	117.7 (7)	C79B—C80B—H80B	120.0
C64B—Sn4—C78B	113.2 (13)	C81B—C80B—H80B	120.0
C71—Sn4—C78B	106.2 (5)	C82B—C81B—C80B	120.0
C64B—Sn4—N7	94.1 (11)	C82B—C81B—H81B	120.0
C78A—Sn4—N7	84.0 (4)	C80B—C81B—H81B	120.0
C71—Sn4—N7	87.45 (17)	C81B—C82B—C83B	120.0
C64A—Sn4—N7	97.1 (4)	C81B—C82B—H82B	120.0
C78B—Sn4—N7	94.6 (4)	C83B—C82B—H82B	120.0
C64B—Sn4—N8	84.1 (12)	C84B—C83B—C82B	120.0
C78A—Sn4—N8	98.3 (4)	C84B—C83B—H83B	120.0
C71—Sn4—N8	92.52 (17)	C82B—C83B—H83B	120.0
C64A—Sn4—N8	80.3 (4)	C83B—C84B—C79B	120.0
C78B—Sn4—N8	88.3 (4)	C83B—C84B—H84B	120.0
N7—Sn4—N8	177.0 (2)	C79B—C84B—H84B	120.0
C92—Sn5—C99	115.65 (19)	C86—C85—Sn5	115.1 (3)
C92—Sn5—C85	121.0 (2)	C86—C85—H85A	108.5
C99—Sn5—C85	123.2 (2)	Sn5—C85—H85A	108.5
C92—Sn5—N9	88.85 (18)	C86—C85—H85B	108.5
C99—Sn5—N9	92.39 (16)	Sn5—C85—H85B	108.5
C85—Sn5—N9	92.81 (15)	H85A—C85—H85B	107.5
C92—Sn5—N10	89.78 (18)	C87—C86—C91	118.5 (4)
C99—Sn5—N10	90.71 (16)	C87—C86—C85	121.8 (4)
C85—Sn5—N10	85.52 (16)	C91—C86—C85	119.7 (5)
N9—Sn5—N10	176.91 (15)	C86—C87—C88	121.5 (4)
C113—Sn6—C106	120.40 (18)	C86—C87—H87	119.2
C113—Sn6—C120	117.9 (2)	C88—C87—H87	119.2
C106—Sn6—C120	121.3 (2)	C89—C88—C87	119.6 (5)
C113—Sn6—N12	87.69 (17)	C89—C88—H88	120.2
C106—Sn6—N12	95.49 (16)	C87—C88—H88	120.2
C120—Sn6—N12	93.43 (17)	C90—C89—C88	119.2 (5)
C113—Sn6—N11	87.83 (17)	C90—C89—H89	120.4
C106—Sn6—N11	90.41 (16)	C88—C89—H89	120.4
C120—Sn6—N11	84.95 (17)	C89—C90—C91	120.7 (4)
N12—Sn6—N11	173.85 (15)	C89—C90—H90	119.6
C127—Sn7—C141	121.6 (2)	C91—C90—H90	119.6
C127—Sn7—C134	110.6 (2)	C90—C91—C86	120.4 (5)
C141—Sn7—C134	127.0 (2)	C90—C91—H91	119.8
C127—Sn7—N13	94.52 (17)	C86—C91—H91	119.8
C141—Sn7—N13	93.53 (16)	C93—C92—Sn5	115.5 (3)
C134—Sn7—N13	90.97 (18)	C93—C92—H92A	108.4
C127—Sn7—N14	86.55 (16)	Sn5—C92—H92A	108.4
C141—Sn7—N14	84.89 (17)	C93—C92—H92B	108.4
C134—Sn7—N14	89.77 (18)	Sn5—C92—H92B	108.4
N13—Sn7—N14	178.39 (16)	H92A—C92—H92B	107.5
C148—Sn8—C163	122.4 (2)	C98—C93—C94	118.5 (6)
C148—Sn8—C156	117.9 (2)	C98—C93—C92	122.4 (5)
C163—Sn8—C156	119.3 (2)	C94—C93—C92	119.2 (6)
C148—Sn8—N15	89.08 (18)	C95—C94—C93	120.1 (8)
C163—Sn8—N15	94.61 (18)	C95—C94—H94	120.0
C156—Sn8—N15	91.93 (18)	C93—C94—H94	120.0
C148—Sn8—N16	90.98 (18)	C96—C95—C94	120.9 (9)
C163—Sn8—N16	85.01 (18)	C96—C95—H95	119.6
C156—Sn8—N16	88.41 (18)	C94—C95—H95	119.6
N15—Sn8—N16	179.58 (15)	C95—C96—C97	121.1 (8)
C201—N1—Sn1	168.7 (4)	C95—C96—H96	119.4
C202—N2—Sn1	157.4 (4)	C97—C96—H96	119.4
C203—N3—Sn2	162.0 (4)	C96—C97—C98	119.1 (9)
C204—N4—Sn2	173.5 (4)	C96—C97—H97	120.4
C205—N5—Sn3	169.1 (4)	C98—C97—H97	120.4
C206—N6—Sn3	164.3 (3)	C93—C98—C97	120.3 (7)
C207—N7—Sn4	155.8 (4)	C93—C98—H98	119.8
C208—N8—Sn4	152.1 (4)	C97—C98—H98	119.8
C209—N9—Sn5	166.3 (4)	C100—C99—Sn5	113.5 (3)
C210—N10—Sn5	165.8 (4)	C100—C99—H99A	108.9
C211—N11—Sn6	155.1 (4)	Sn5—C99—H99A	108.9
C212—N12—Sn6	170.9 (4)	C100—C99—H99B	108.9
C213—N13—Sn7	162.0 (4)	Sn5—C99—H99B	108.9
C214—N14—Sn7	154.2 (4)	H99A—C99—H99B	107.7
C215—N15—Sn8	158.5 (4)	C101—C100—C105	116.8 (5)
C216—N16—Sn8	170.3 (4)	C101—C100—C99	121.8 (5)
C2—C1—Sn1	107.9 (3)	C105—C100—C99	121.4 (4)
C2—C1—H1A	110.1	C102—C101—C100	120.5 (6)
Sn1—C1—H1A	110.1	C102—C101—H10A	119.7
C2—C1—H1B	110.1	C100—C101—H10A	119.7
Sn1—C1—H1B	110.1	C101—C102—C103	121.5 (6)
H1A—C1—H1B	108.4	C101—C102—H10B	119.3
C3—C2—C7	117.5 (6)	C103—C102—H10B	119.3
C3—C2—C1	121.2 (5)	C104—C103—C102	118.6 (6)
C7—C2—C1	121.3 (6)	C104—C103—H10C	120.7
C2—C3—C4	120.0 (7)	C102—C103—H10C	120.7
C2—C3—H3	120.0	C103—C104—C105	121.0 (6)
C4—C3—H3	120.0	C103—C104—H10D	119.5
C5—C4—C3	119.2 (7)	C105—C104—H10D	119.5
C5—C4—H4	120.4	C104—C105—C100	121.6 (5)
C3—C4—H4	120.4	C104—C105—H10E	119.2
C6—C5—C4	120.3 (8)	C100—C105—H10E	119.2
C6—C5—H5	119.9	C107—C106—Sn6	112.9 (3)
C4—C5—H5	119.9	C107—C106—H10F	109.0
C5—C6—C7	121.1 (8)	Sn6—C106—H10F	109.0
C5—C6—H6	119.4	C107—C106—H10G	109.0
C7—C6—H6	119.4	Sn6—C106—H10G	109.0
C6—C7—C2	121.9 (8)	H10F—C106—H10G	107.8
C6—C7—H7	119.0	C108—C107—C112	118.8 (5)
C2—C7—H7	119.0	C108—C107—C106	121.6 (5)
C14—C8—Sn1	114.7 (3)	C112—C107—C106	119.5 (5)
C14—C8—H8A	108.6	C107—C108—C109	120.7 (5)
Sn1—C8—H8A	108.6	C107—C108—H10H	119.6
C14—C8—H8B	108.6	C109—C108—H10H	119.6
Sn1—C8—H8B	108.6	C110—C109—C108	120.2 (5)
H8A—C8—H8B	107.6	C110—C109—H10I	119.9
C14—C9—C10	121.1 (4)	C108—C109—H10I	119.9
C14—C9—H9	119.4	C111—C110—C109	119.6 (5)
C10—C9—H9	119.4	C111—C110—H11A	120.2
C11—C10—C9	120.4 (5)	C109—C110—H11A	120.2
C11—C10—H10	119.8	C110—C111—C112	119.9 (5)
C9—C10—H10	119.8	C110—C111—H11B	120.0
C12—C11—C10	119.3 (5)	C112—C111—H11B	120.0
C12—C11—H11	120.3	C107—C112—C111	120.7 (5)
C10—C11—H11	120.3	C107—C112—H11C	119.6
C11—C12—C13	120.5 (5)	C111—C112—H11C	119.6
C11—C12—H12	119.7	C114—C113—Sn6	111.1 (3)
C13—C12—H12	119.7	C114—C113—H11D	109.4
C12—C13—C14	120.8 (5)	Sn6—C113—H11D	109.4
C12—C13—H13	119.6	C114—C113—H11E	109.4
C14—C13—H13	119.6	Sn6—C113—H11E	109.4
C9—C14—C13	117.8 (5)	H11D—C113—H11E	108.0
C9—C14—C8	122.1 (4)	C115—C114—C119	118.2 (5)
C13—C14—C8	120.1 (4)	C115—C114—C113	120.7 (4)
C16—C15—Sn1	115.7 (4)	C119—C114—C113	121.1 (5)
C16—C15—H15A	108.3	C114—C115—C116	123.1 (6)
Sn1—C15—H15A	108.3	C114—C115—H11F	118.4
C16—C15—H15B	108.3	C116—C115—H11F	118.4
Sn1—C15—H15B	108.3	C117—C116—C115	118.1 (7)
H15A—C15—H15B	107.4	C117—C116—H11G	120.9
C17—C16—C21	116.9 (6)	C115—C116—H11G	120.9
C17—C16—C15	122.3 (6)	C118—C117—C116	121.3 (7)
C21—C16—C15	120.8 (5)	C118—C117—H11H	119.3
C18—C17—C16	122.5 (7)	C116—C117—H11H	119.3
C18—C17—H17	118.8	C117—C118—C119	120.8 (6)
C16—C17—H17	118.8	C117—C118—H11I	119.6
C19—C18—C17	120.1 (8)	C119—C118—H11I	119.6
C19—C18—H18	120.0	C114—C119—C118	118.4 (6)
C17—C18—H18	120.0	C114—C119—H11J	120.8
C18—C19—C20	120.0 (8)	C118—C119—H11J	120.8
C18—C19—H19	120.0	C121—C120—Sn6	120.2 (3)
C20—C19—H19	120.0	C121—C120—H12A	107.3
C19—C20—C21	121.0 (8)	Sn6—C120—H12A	107.3
C19—C20—H20	119.5	C121—C120—H12B	107.3
C21—C20—H20	119.5	Sn6—C120—H12B	107.3
C16—C21—C20	119.5 (7)	H12A—C120—H12B	106.9
C16—C21—H21	120.3	C122—C121—C126	117.0 (5)
C20—C21—H21	120.3	C122—C121—C120	121.6 (5)
C23—C22—Sn2	111.5 (3)	C126—C121—C120	121.3 (5)
C23—C22—H22A	109.3	C123—C122—C121	121.2 (6)
Sn2—C22—H22A	109.3	C123—C122—H12C	119.4
C23—C22—H22B	109.3	C121—C122—H12C	119.4
Sn2—C22—H22B	109.3	C124—C123—C122	121.3 (7)
H22A—C22—H22B	108.0	C124—C123—H12D	119.3
C24—C23—C28	117.9 (6)	C122—C123—H12D	119.3
C24—C23—C22	121.3 (5)	C123—C124—C125	118.9 (6)
C28—C23—C22	120.8 (5)	C123—C124—H12E	120.5
C23—C24—C25	121.1 (6)	C125—C124—H12E	120.5
C23—C24—H24	119.4	C126—C125—C124	120.5 (6)
C25—C24—H24	119.4	C126—C125—H12F	119.8
C26—C25—C24	120.1 (7)	C124—C125—H12F	119.8
C26—C25—H25	119.9	C125—C126—C121	121.2 (6)
C24—C25—H25	119.9	C125—C126—H12G	119.4
C27—C26—C25	118.8 (7)	C121—C126—H12G	119.4
C27—C26—H26	120.6	C128—C127—Sn7	118.8 (3)
C25—C26—H26	120.6	C128—C127—H12H	107.6
C26—C27—C28	122.2 (8)	Sn7—C127—H12H	107.6
C26—C27—H27	118.9	C128—C127—H12I	107.6
C28—C27—H27	118.9	Sn7—C127—H12I	107.6
C27—C28—C23	119.9 (6)	H12H—C127—H12I	107.1
C27—C28—H28	120.0	C133—C128—C129	116.8 (5)
C23—C28—H28	120.0	C133—C128—C127	120.5 (5)
C37—C29—Sn2	110.6 (3)	C129—C128—C127	122.7 (5)
C37—C29—H29A	109.5	C128—C129—C130	121.7 (6)
Sn2—C29—H29A	109.5	C128—C129—H12J	119.1
C37—C29—H29B	109.5	C130—C129—H12J	119.1
Sn2—C29—H29B	109.5	C131—C130—C129	119.7 (6)
H29A—C29—H29B	108.1	C131—C130—H13A	120.1
C31—C30—C35	117.3 (5)	C129—C130—H13A	120.1
C31—C30—C36	121.0 (5)	C132—C131—C130	119.4 (6)
C35—C30—C36	121.5 (5)	C132—C131—H13B	120.3
C32—C31—C30	122.1 (5)	C130—C131—H13B	120.3
C32—C31—H31	118.9	C131—C132—C133	120.4 (6)
C30—C31—H31	118.9	C131—C132—H13C	119.8
C31—C32—C33	119.9 (6)	C133—C132—H13C	119.8
C31—C32—H32	120.1	C128—C133—C132	121.9 (6)
C33—C32—H32	120.1	C128—C133—H13D	119.1
C34—C33—C32	119.3 (6)	C132—C133—H13D	119.1
C34—C33—H33	120.4	C135—C134—Sn7	107.8 (4)
C32—C33—H33	120.4	C135—C134—H13E	110.2
C33—C34—C35	120.7 (5)	Sn7—C134—H13E	110.2
C33—C34—H34	119.6	C135—C134—H13F	110.2
C35—C34—H34	119.6	Sn7—C134—H13F	110.2
C34—C35—C30	120.7 (6)	H13E—C134—H13F	108.5
C34—C35—H35	119.7	C136—C135—C140	115.2 (6)
C30—C35—H35	119.7	C136—C135—C134	122.2 (6)
C30—C36—Sn2	115.0 (3)	C140—C135—C134	122.5 (5)
C30—C36—H36A	108.5	C135—C136—C137	121.6 (8)
Sn2—C36—H36A	108.5	C135—C136—H13G	119.2
C30—C36—H36B	108.5	C137—C136—H13G	119.2
Sn2—C36—H36B	108.5	C138—C137—C136	120.5 (9)
H36A—C36—H36B	107.5	C138—C137—H13H	119.8
C42—C37—C38	117.9 (4)	C136—C137—H13H	119.8
C42—C37—C29	120.4 (4)	C137—C138—C139	120.2 (9)
C38—C37—C29	121.7 (4)	C137—C138—H13I	119.9
C39—C38—C37	120.6 (5)	C139—C138—H13I	119.9
C39—C38—H38	119.7	C138—C139—C140	119.2 (8)
C37—C38—H38	119.7	C138—C139—H13J	120.4
C40—C39—C38	120.8 (5)	C140—C139—H13J	120.4
C40—C39—H39	119.6	C139—C140—C135	123.4 (7)
C38—C39—H39	119.6	C139—C140—H14A	118.3
C39—C40—C41	119.2 (5)	C135—C140—H14A	118.3
C39—C40—H40	120.4	C142—C141—Sn7	116.8 (3)
C41—C40—H40	120.4	C142—C141—H14B	108.1
C42—C41—C40	119.9 (5)	Sn7—C141—H14B	108.1
C42—C41—H41	120.1	C142—C141—H14C	108.1
C40—C41—H41	120.1	Sn7—C141—H14C	108.1
C41—C42—C37	121.6 (5)	H14B—C141—H14C	107.3
C41—C42—H42	119.2	C143—C142—C147	117.7 (5)
C37—C42—H42	119.2	C143—C142—C141	122.6 (5)
C44—C43—Sn3	115.1 (3)	C147—C142—C141	119.7 (5)
C44—C43—H43A	108.5	C142—C143—C144	119.4 (6)
Sn3—C43—H43A	108.5	C142—C143—H14D	120.3
C44—C43—H43B	108.5	C144—C143—H14D	120.3
Sn3—C43—H43B	108.5	C145—C144—C143	119.4 (6)
H43A—C43—H43B	107.5	C145—C144—H14E	120.3
C45—C44—C49	120.0	C143—C144—H14E	120.3
C45—C44—C43	120.7 (3)	C146—C145—C144	121.6 (6)
C49—C44—C43	119.3 (3)	C146—C145—H14F	119.2
C44—C45—C46	120.0	C144—C145—H14F	119.2
C44—C45—H45	120.0	C145—C146—C147	119.9 (7)
C46—C45—H45	120.0	C145—C146—H14G	120.1
C47—C46—C45	120.0	C147—C146—H14G	120.1
C47—C46—H46	120.0	C146—C147—C142	122.1 (6)
C45—C46—H46	120.0	C146—C147—H14H	119.0
C46—C47—C48	120.0	C142—C147—H14H	119.0
C46—C47—H47	120.0	C149—C148—Sn8	112.8 (3)
C48—C47—H47	120.0	C149—C148—H14I	109.0
C47—C48—C49	120.0	Sn8—C148—H14I	109.0
C47—C48—H48	120.0	C149—C148—H14J	109.0
C49—C48—H48	120.0	Sn8—C148—H14J	109.0
C48—C49—C44	120.0	H14I—C148—H14J	107.8
C48—C49—H49	120.0	C151—C149—C155	116.7 (6)
C44—C49—H49	120.0	C151—C149—C148	121.7 (5)
C51—C50—Sn3	111.4 (3)	C155—C149—C148	121.5 (5)
C51—C50—H50A	109.4	C149—C151—C152	120.6 (7)
Sn3—C50—H50A	109.4	C149—C151—H15C	119.7
C51—C50—H50B	109.4	C152—C151—H15C	119.7
Sn3—C50—H50B	109.4	C153—C152—C151	120.3 (7)
H50A—C50—H50B	108.0	C153—C152—H15D	119.8
C56—C51—C52	118.1 (4)	C151—C152—H15D	119.8
C56—C51—C50	121.0 (4)	C154—C153—C152	119.5 (7)
C52—C51—C50	120.6 (5)	C154—C153—H15E	120.3
C51—C52—C53	120.2 (5)	C152—C153—H15E	120.3
C51—C52—H52	119.9	C153—C154—C155	120.1 (7)
C53—C52—H52	119.9	C153—C154—H15F	120.0
C54—C53—C52	120.7 (5)	C155—C154—H15F	120.0
C54—C53—H53	119.6	C154—C155—C149	122.7 (6)
C52—C53—H53	119.6	C154—C155—H15G	118.7
C53—C54—C55	119.7 (5)	C149—C155—H15G	118.7
C53—C54—H54	120.1	C157—C156—Sn8	111.5 (3)
C55—C54—H54	120.1	C157—C156—H15H	109.3
C54—C55—C56	119.2 (5)	Sn8—C156—H15H	109.3
C54—C55—H55	120.4	C157—C156—H15I	109.3
C56—C55—H55	120.4	Sn8—C156—H15I	109.3
C51—C56—C55	121.9 (5)	H15H—C156—H15I	108.0
C51—C56—H56	119.1	C158—C157—C162	116.6 (6)
C55—C56—H56	119.1	C158—C157—C156	122.1 (6)
C58—C57—Sn3	109.7 (3)	C162—C157—C156	121.3 (5)
C58—C57—H57A	109.7	C157—C158—C159	121.5 (7)
Sn3—C57—H57A	109.7	C157—C158—H15K	119.2
C58—C57—H57B	109.7	C159—C158—H15K	119.2
Sn3—C57—H57B	109.7	C160—C159—C158	120.9 (7)
H57A—C57—H57B	108.2	C160—C159—H15L	119.6
C63—C58—C59	118.1 (5)	C158—C159—H15L	119.6
C63—C58—C57	120.8 (4)	C159—C160—C161	118.4 (8)
C59—C58—C57	121.0 (5)	C159—C160—H16A	120.8
C60—C59—C58	120.7 (6)	C161—C160—H16A	120.8
C60—C59—H59	119.6	C162—C161—C160	121.2 (8)
C58—C59—H59	119.6	C162—C161—H16B	119.4
C61—C60—C59	119.6 (6)	C160—C161—H16B	119.4
C61—C60—H60	120.2	C161—C162—C157	121.4 (7)
C59—C60—H60	120.2	C161—C162—H16C	119.3
C60—C61—C62	121.0 (6)	C157—C162—H16C	119.3
C60—C61—H61	119.5	C164—C163—Sn8	116.0 (4)
C62—C61—H61	119.5	C164—C163—H16D	108.3
C61—C62—C63	118.6 (6)	Sn8—C163—H16D	108.3
C61—C62—H62	120.7	C164—C163—H16E	108.3
C63—C62—H62	120.7	Sn8—C163—H16E	108.3
C58—C63—C62	121.8 (5)	H16D—C163—H16E	107.4
C58—C63—H63	119.1	C165—C164—C169	117.1 (6)
C62—C63—H63	119.1	C165—C164—C163	120.6 (5)
C65—C64A—Sn4	118.4 (7)	C169—C164—C163	122.3 (6)
C65—C64A—H64A	107.7	C166—C165—C164	121.9 (6)
Sn4—C64A—H64A	107.7	C166—C165—H16F	119.0
C65—C64A—H64B	107.7	C164—C165—H16F	119.0
Sn4—C64A—H64B	107.7	C165—C166—C167	119.4 (7)
H64A—C64A—H64B	107.1	C165—C166—H16G	120.3
C65—C64B—Sn4	117.0 (10)	C167—C166—H16G	120.3
C65—C64B—H64C	108.1	C168—C167—C166	119.2 (7)
Sn4—C64B—H64C	108.1	C168—C167—H16H	120.4
C65—C64B—H64D	108.1	C166—C167—H16H	120.4
Sn4—C64B—H64D	108.1	C167—C168—C169	121.5 (6)
H64C—C64B—H64D	107.3	C167—C168—H16I	119.3
C66—C65—C70	120.0	C169—C168—H16I	119.3
C66—C65—C64A	109.1 (12)	C168—C169—C164	120.7 (7)
C70—C65—C64A	130.2 (12)	C168—C169—H16J	119.6
C66—C65—C64B	138.3 (17)	C164—C169—H16J	119.6
C70—C65—C64B	101.7 (17)	N1—C201—Ni4	177.5 (4)
C65—C66—C67	120.0	N2—C202—Ni1	175.7 (5)
C65—C66—H66	120.0	N3—C203—Ni1	173.1 (4)
C67—C66—H66	120.0	N4—C204—Ni4^v^	177.4 (5)
C68—C67—C66	120.0	N5—C205—Ni1	177.0 (4)
C68—C67—H67	120.0	N6—C206—Ni2	176.4 (4)
C66—C67—H67	120.0	N7—C207—Ni1	175.1 (5)
C67—C68—C69	120.0	N8—C208—Ni2^ii^	177.2 (4)
C67—C68—H68	120.0	N9—C209—Ni2	179.6 (4)
C69—C68—H68	120.0	N10—C210—Ni3	175.4 (5)
C70—C69—C68	120.0	N11—C211—Ni3^i^	174.9 (4)
C70—C69—H69	120.0	N12—C212—Ni2	179.1 (4)
C68—C69—H69	120.0	N13—C213—Ni3	176.8 (4)
C69—C70—C65	120.0	N14—C214—Ni4^vi^	176.7 (4)
C69—C70—H70	120.0	N15—C215—Ni3	173.7 (4)
C65—C70—H70	120.0	N16—C216—Ni4^iv^	179.1 (5)
C72—C71—Sn4	118.0 (3)		
			
Sn1—C1—C2—C3	94.2 (5)	C78B—C79B—C80B—C81B	177.9 (12)
Sn1—C1—C2—C7	−83.4 (6)	C79B—C80B—C81B—C82B	0.0
C7—C2—C3—C4	0.9 (9)	C80B—C81B—C82B—C83B	0.0
C1—C2—C3—C4	−176.8 (5)	C81B—C82B—C83B—C84B	0.0
C2—C3—C4—C5	−0.3 (10)	C82B—C83B—C84B—C79B	0.0
C3—C4—C5—C6	0.0 (11)	C80B—C79B—C84B—C83B	0.0
C4—C5—C6—C7	−0.3 (12)	C78B—C79B—C84B—C83B	−178.1 (11)
C5—C6—C7—C2	1.0 (11)	Sn5—C85—C86—C87	87.5 (5)
C3—C2—C7—C6	−1.3 (9)	Sn5—C85—C86—C91	−91.3 (5)
C1—C2—C7—C6	176.4 (6)	C91—C86—C87—C88	0.6 (7)
C14—C9—C10—C11	−1.4 (9)	C85—C86—C87—C88	−178.2 (4)
C9—C10—C11—C12	0.7 (10)	C86—C87—C88—C89	0.3 (7)
C10—C11—C12—C13	0.4 (11)	C87—C88—C89—C90	−0.7 (7)
C11—C12—C13—C14	−0.9 (11)	C88—C89—C90—C91	0.3 (7)
C10—C9—C14—C13	0.9 (8)	C89—C90—C91—C86	0.6 (7)
C10—C9—C14—C8	179.2 (5)	C87—C86—C91—C90	−1.0 (7)
C12—C13—C14—C9	0.2 (9)	C85—C86—C91—C90	177.8 (4)
C12—C13—C14—C8	−178.1 (6)	Sn5—C92—C93—C98	80.2 (6)
Sn1—C8—C14—C9	81.8 (5)	Sn5—C92—C93—C94	−100.7 (5)
Sn1—C8—C14—C13	−100.0 (5)	C98—C93—C94—C95	0.7 (9)
Sn1—C15—C16—C17	−96.6 (6)	C92—C93—C94—C95	−178.5 (6)
Sn1—C15—C16—C21	82.4 (6)	C93—C94—C95—C96	−1.8 (11)
C21—C16—C17—C18	1.7 (10)	C94—C95—C96—C97	1.6 (13)
C15—C16—C17—C18	−179.2 (6)	C95—C96—C97—C98	−0.3 (12)
C16—C17—C18—C19	−3.8 (13)	C94—C93—C98—C97	0.6 (9)
C17—C18—C19—C20	3.3 (14)	C92—C93—C98—C97	179.7 (5)
C18—C19—C20—C21	−0.9 (14)	C96—C97—C98—C93	−0.8 (11)
C17—C16—C21—C20	0.7 (9)	Sn5—C99—C100—C101	−92.6 (5)
C15—C16—C21—C20	−178.4 (6)	Sn5—C99—C100—C105	87.8 (5)
C19—C20—C21—C16	−1.1 (12)	C105—C100—C101—C102	0.8 (7)
Sn2—C22—C23—C24	98.5 (5)	C99—C100—C101—C102	−178.8 (5)
Sn2—C22—C23—C28	−80.5 (6)	C100—C101—C102—C103	0.4 (9)
C28—C23—C24—C25	−0.3 (8)	C101—C102—C103—C104	−1.3 (9)
C22—C23—C24—C25	−179.3 (5)	C102—C103—C104—C105	0.9 (9)
C23—C24—C25—C26	−0.2 (9)	C103—C104—C105—C100	0.4 (8)
C24—C25—C26—C27	0.5 (11)	C101—C100—C105—C104	−1.2 (7)
C25—C26—C27—C28	−0.3 (13)	C99—C100—C105—C104	178.4 (5)
C26—C27—C28—C23	−0.2 (13)	Sn6—C106—C107—C108	80.0 (5)
C24—C23—C28—C27	0.5 (10)	Sn6—C106—C107—C112	−99.5 (5)
C22—C23—C28—C27	179.5 (6)	C112—C107—C108—C109	−0.8 (7)
C35—C30—C31—C32	1.2 (9)	C106—C107—C108—C109	179.7 (5)
C36—C30—C31—C32	−174.8 (6)	C107—C108—C109—C110	0.7 (8)
C30—C31—C32—C33	−1.2 (11)	C108—C109—C110—C111	−0.6 (9)
C31—C32—C33—C34	0.3 (11)	C109—C110—C111—C112	0.5 (9)
C32—C33—C34—C35	0.7 (11)	C108—C107—C112—C111	0.8 (7)
C33—C34—C35—C30	−0.8 (10)	C106—C107—C112—C111	−179.7 (4)
C31—C30—C35—C34	−0.1 (9)	C110—C111—C112—C107	−0.6 (8)
C36—C30—C35—C34	175.8 (5)	Sn6—C113—C114—C115	−92.8 (5)
C31—C30—C36—Sn2	87.0 (6)	Sn6—C113—C114—C119	86.5 (5)
C35—C30—C36—Sn2	−88.8 (6)	C119—C114—C115—C116	−0.1 (9)
Sn2—C29—C37—C42	89.2 (5)	C113—C114—C115—C116	179.2 (6)
Sn2—C29—C37—C38	−90.0 (5)	C114—C115—C116—C117	−0.6 (10)
C42—C37—C38—C39	−0.7 (7)	C115—C116—C117—C118	0.6 (12)
C29—C37—C38—C39	178.5 (4)	C116—C117—C118—C119	0.1 (13)
C37—C38—C39—C40	0.7 (7)	C115—C114—C119—C118	0.7 (9)
C38—C39—C40—C41	0.5 (8)	C113—C114—C119—C118	−178.6 (6)
C39—C40—C41—C42	−1.6 (8)	C117—C118—C119—C114	−0.7 (11)
C40—C41—C42—C37	1.6 (8)	Sn6—C120—C121—C122	−78.8 (6)
C38—C37—C42—C41	−0.4 (8)	Sn6—C120—C121—C126	103.6 (5)
C29—C37—C42—C41	−179.6 (5)	C126—C121—C122—C123	0.6 (7)
Sn3—C43—C44—C45	73.8 (4)	C120—C121—C122—C123	−177.0 (4)
Sn3—C43—C44—C49	−108.4 (3)	C121—C122—C123—C124	−0.8 (8)
C49—C44—C45—C46	0.0	C122—C123—C124—C125	0.4 (8)
C43—C44—C45—C46	177.8 (4)	C123—C124—C125—C126	0.2 (8)
C44—C45—C46—C47	0.0	C124—C125—C126—C121	−0.3 (8)
C45—C46—C47—C48	0.0	C122—C121—C126—C125	−0.1 (7)
C46—C47—C48—C49	0.0	C120—C121—C126—C125	177.5 (4)
C47—C48—C49—C44	0.0	Sn7—C127—C128—C133	128.0 (5)
C45—C44—C49—C48	0.0	Sn7—C127—C128—C129	−53.9 (6)
C43—C44—C49—C48	−177.8 (4)	C133—C128—C129—C130	−0.4 (9)
Sn3—C50—C51—C56	−71.9 (5)	C127—C128—C129—C130	−178.5 (6)
Sn3—C50—C51—C52	103.0 (5)	C128—C129—C130—C131	2.0 (10)
C56—C51—C52—C53	1.9 (8)	C129—C130—C131—C132	−1.7 (10)
C50—C51—C52—C53	−173.2 (5)	C130—C131—C132—C133	−0.2 (11)
C51—C52—C53—C54	0.3 (8)	C129—C128—C133—C132	−1.5 (9)
C52—C53—C54—C55	−1.2 (9)	C127—C128—C133—C132	176.6 (6)
C53—C54—C55—C56	0.0 (9)	C131—C132—C133—C128	1.9 (11)
C52—C51—C56—C55	−3.1 (8)	Sn7—C134—C135—C136	92.1 (6)
C50—C51—C56—C55	171.9 (5)	Sn7—C134—C135—C140	−82.9 (5)
C54—C55—C56—C51	2.2 (9)	C140—C135—C136—C137	1.7 (11)
Sn3—C57—C58—C63	97.3 (5)	C134—C135—C136—C137	−173.7 (7)
Sn3—C57—C58—C59	−78.8 (5)	C135—C136—C137—C138	−1.9 (15)
C63—C58—C59—C60	2.1 (8)	C136—C137—C138—C139	1.6 (16)
C57—C58—C59—C60	178.4 (5)	C137—C138—C139—C140	−1.0 (14)
C58—C59—C60—C61	−0.7 (10)	C138—C139—C140—C135	0.9 (11)
C59—C60—C61—C62	−1.7 (12)	C136—C135—C140—C139	−1.2 (9)
C60—C61—C62—C63	2.6 (12)	C134—C135—C140—C139	174.2 (6)
C59—C58—C63—C62	−1.2 (8)	Sn7—C141—C142—C143	−99.2 (6)
C57—C58—C63—C62	−177.5 (6)	Sn7—C141—C142—C147	79.1 (6)
C61—C62—C63—C58	−1.1 (11)	C147—C142—C143—C144	0.0 (9)
Sn4—C64A—C65—C66	−106.1 (15)	C141—C142—C143—C144	178.3 (6)
Sn4—C64A—C65—C70	83.6 (14)	C142—C143—C144—C145	−0.7 (10)
Sn4—C64B—C65—C66	−58 (4)	C143—C144—C145—C146	0.7 (12)
Sn4—C64B—C65—C70	122 (3)	C144—C145—C146—C147	0.0 (12)
C70—C65—C66—C67	0.0	C145—C146—C147—C142	−0.7 (10)
C64A—C65—C66—C67	−171.5 (5)	C143—C142—C147—C146	0.7 (9)
C64B—C65—C66—C67	179.7 (18)	C141—C142—C147—C146	−177.7 (6)
C65—C66—C67—C68	0.0	Sn8—C148—C149—C151	−108.0 (5)
C66—C67—C68—C69	0.0	Sn8—C148—C149—C155	71.3 (6)
C67—C68—C69—C70	0.0	C155—C149—C151—C152	2.8 (9)
C68—C69—C70—C65	0.0	C148—C149—C151—C152	−177.9 (6)
C66—C65—C70—C69	0.0	C149—C151—C152—C153	−1.5 (12)
C64A—C65—C70—C69	169.5 (7)	C151—C152—C153—C154	0.1 (12)
C64B—C65—C70—C69	−179.8 (12)	C152—C153—C154—C155	0.0 (12)
Sn4—C71—C72—C73	−84.9 (5)	C153—C154—C155—C149	1.5 (11)
Sn4—C71—C72—C77	97.0 (5)	C151—C149—C155—C154	−2.9 (9)
C77—C72—C73—C74	2.7 (8)	C148—C149—C155—C154	177.8 (6)
C71—C72—C73—C74	−175.5 (5)	Sn8—C156—C157—C158	82.9 (5)
C72—C73—C74—C75	−1.8 (8)	Sn8—C156—C157—C162	−94.6 (5)
C73—C74—C75—C76	−0.1 (9)	C162—C157—C158—C159	−0.3 (9)
C74—C75—C76—C77	0.9 (9)	C156—C157—C158—C159	−177.9 (6)
C75—C76—C77—C72	0.0 (9)	C157—C158—C159—C160	0.2 (11)
C73—C72—C77—C76	−1.8 (8)	C158—C159—C160—C161	0.1 (13)
C71—C72—C77—C76	176.4 (5)	C159—C160—C161—C162	−0.2 (12)
Sn4—C78A—C79A—C80A	96.5 (12)	C160—C161—C162—C157	0.1 (11)
Sn4—C78A—C79A—C84A	−75.7 (12)	C158—C157—C162—C161	0.2 (9)
C84A—C79A—C80A—C81A	0.0	C156—C157—C162—C161	177.8 (6)
C78A—C79A—C80A—C81A	−171.9 (13)	Sn8—C163—C164—C165	−84.9 (6)
C79A—C80A—C81A—C82A	0.0	Sn8—C163—C164—C169	95.3 (6)
C80A—C81A—C82A—C83A	0.0	C169—C164—C165—C166	−1.9 (9)
C81A—C82A—C83A—C84A	0.0	C163—C164—C165—C166	178.3 (6)
C82A—C83A—C84A—C79A	0.0	C164—C165—C166—C167	0.5 (11)
C80A—C79A—C84A—C83A	0.0	C165—C166—C167—C168	1.9 (11)
C78A—C79A—C84A—C83A	172.5 (11)	C166—C167—C168—C169	−2.9 (11)
Sn4—C78B—C79B—C80B	87.2 (13)	C167—C168—C169—C164	1.5 (10)
Sn4—C78B—C79B—C84B	−94.8 (12)	C165—C164—C169—C168	0.9 (9)
C84B—C79B—C80B—C81B	0.0	C163—C164—C169—C168	−179.3 (6)

Symmetry codes: (i) *x*−1, *y*, *z*; (ii) *x*+1, *y*, *z*; (iii) *x*+1, *y*, *z*−1; (iv) −*x*+2, −*y*+1, −*z*; (v) −*x*+1, −*y*, −*z*−1; (vi) *x*−1, *y*, *z*+1.

### Poly[hexabenzyltetra-µ-cyanato-nickel(II)ditin(IV)] Hydrogen-bond geometry (Å, º)

*Cg*9, *Cg*13, *Cg*15, *Cg*18, and *Cg*23 are the centroids of rings C58–C63, C86–C91, C100–C105, C121–C126 and C157–C162, respectively.

**Table:**

*D*—H···*A*	*D*—H	H···*A*	*D*···*A*	*D*—H···*A*
C117—H11*H*···*Cg*9^i^	0.95	2.85	3.77 (2)	163
C165—H16*F*···*Cg*23	0.95	2.70	3.505 (7)	142
C40—H40···*Cg*13^vii^	0.95	2.63	3.502 (6)	153
C76—H76···*Cg*15	0.95	2.78	3.377 (6)	122
C87—H87···*Cg*15	0.95	2.97	3.537 (5)	120
C90—H90···*Cg*18	0.99	2.68	3.584 (5)	159

Symmetry codes: (i) *x*−1, *y*, *z*; (vii) −*x*, −*y*, −*z*.
