# Supplementary material for: A PtS-like 42.84 topology arising from the self-assembly of square-planar nodes via organotin units: synthesis and crystal structure of the metal–organic framework {[(PhCH2)3Sn]2Ni(CN)4}n
Source: Acta Crystallogr E Crystallogr Commun. 2026 Mar 17;82(Pt 4):357–61. doi: 10.1107/S2056989026002392 (PMC13055984; doi:10.1107/S2056989026002392)
Supplement: Supplementary file 3 [file e-82-00357-sup3.pdf]

Table S1. Selected bond lengths and bond angles (Å, °) for compound (I)

Bond	Bond length (Å)
Sn1—N1	2.373 (4)
Sn1—N2	2.274 (4)
Sn2—N3	2.273 (4)
Sn2—N4	2.358 (4)
Sn3—N5	2.322 (4)
Sn3—N6	2.330 (3)
Sn4—N7	2.311 (5)
Sn4—N8	2.342 (4)
Sn5—N9	2.303 (4)
Sn5—N10	2.335 (4)
Sn6—N11	2.340 (4)
Sn6—N12	2.296 (4)
Sn7—N13	2.261 (4)
Sn7—N14	2.393 (4)
Sn8—N15	2.244 (4)
Sn8—N16	2.387 (4)
Ni1—C202	1.852 (5)
Ni1—C203	1.848 (4)
Ni1—C205	1.854 (4)
Ni1—C207	1.863 (5)
Ni2—C206	1.862 (4)
Ni2—C209	1.854 (4)
Ni2—C212	1.856 (5)
Ni2—C208^{i}	1.860 (4)
Ni3—C210	1.856 (5)
Ni3—C213	1.858 (4)

Ni3—C215	1.841 (4)
Ni3—C211^{ii}	1.846 (4)
Ni4—C201	1.855 (5)
Ni4—C214^{iii}	1.848 (5)
Ni4—C204^{iv}	1.857 (4)
Ni4—C216^{v}	1.855 (4)

**Angle**	**Bond angle (°)**
N1—Sn1—N2	177.15 (15)
C1—Sn1—C15	127.1 (2)
N3—Sn2—N4	178.14 (15)
C22—Sn2—C36	120.4 (2)
N5—Sn3—N6	177.09 (14)
C43—Sn3—C50	127.10 (18)
N7—Sn4—N8	177.02 (19)
C71—Sn4—C78A	127.0 (5)
N9—Sn5—N10	176.94 (14)
C85—Sn5—C99	123.2 (2)
N11—Sn6—N12	173.85 (15)
C106—Sn6—C120	121.2 (2)
N13—Sn7—N14	178.41 (15)
C134—Sn7—C141	127.0 (2)
N15—Sn8—N16	179.59 (15)
C148—Sn8—C163	122.5 (2)
C202—Ni1—C205	172.66 (19)
C203—Ni1—C207	172.7 (2)
C206—Ni2—C212	177.2 (2)
C208^{i}—Ni2—C209	178.82 (19)
C210—Ni3—C215	173.03 (19)

C211^{ii}—Ni3—C213	174.19 (19)
C201—Ni4—C214^{iii}	179.10 (19)
C204^{iv}—Ni4—C216^{v}	177.84 (19)
Sn1—N1—C201	168.7 (4)
Sn1—N2—C202	157.4 (4)
Sn2—N3—C203	162.0 (4)
Sn2—N4—C204	173.4 (4)
Sn3—N5—C205	169.0 (4)
Sn3—N6—C206	164.3 (3)
Sn4—N7—C207	155.8 (4)
Sn4—N8—C208	152.1 (4)
Sn5—N9—C209	166.2 (3)
Sn5—N10—C210	165.8 (4)
Sn6—N11—C211	155.1 (4)
Sn6—N12—C212	170.9 (4)
Sn7—N13—C213	162.0 (4)
Sn7—N14—C214	154.2 (4)
Sn8—N15—C215	158.6 (4)
Sn8—N16—C216	170.3 (4)

Symmetry codes: (i) *x*−1, *y*, *z*; (ii) *x*+1, *y*, *z*; (iii) *x*+1, *y*, *z*−1; (iv) −*x*+1, −*y*, −*z*−1; (v) −*x*+2, −*y*+1, −*z*; (vi) *x*−1, *y*, *z*+1.

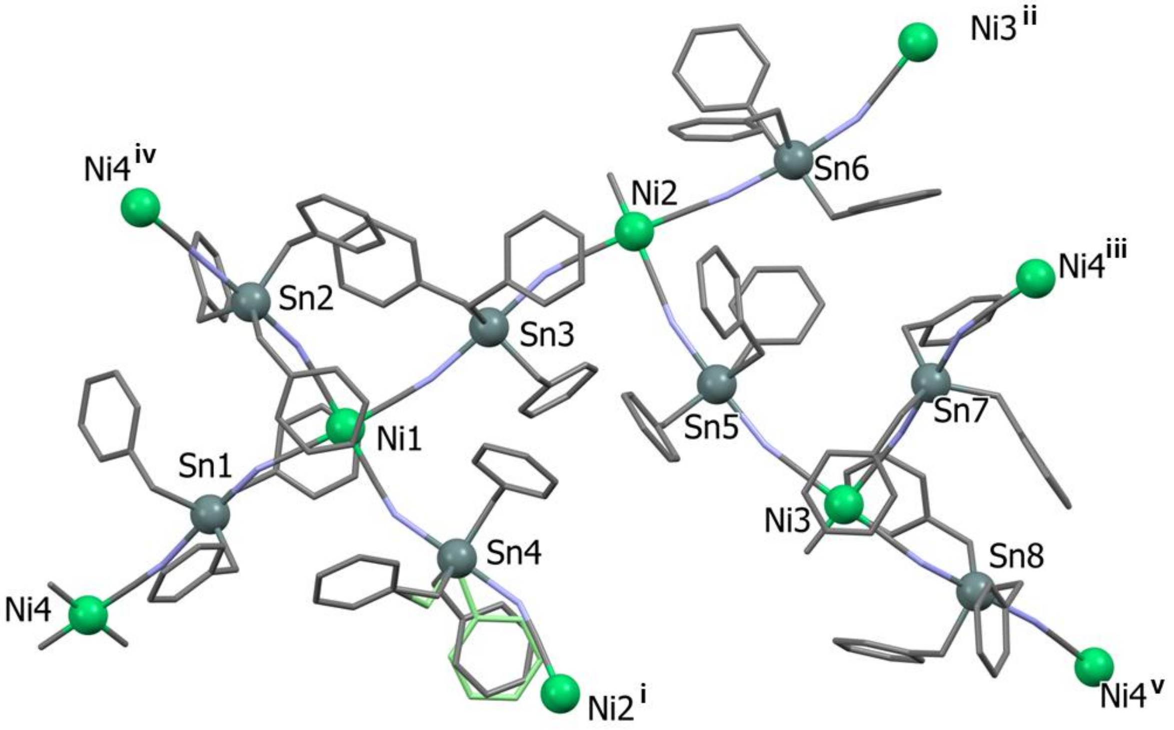

Figure S1. A view of the molecular structure of the asymmetric unit of compound (**I**). For clarity, only the metal atoms have been labelled, and the positionally disordered atoms are shown in pale green. [Symmetry codes: (i) 1+x, y, z; (ii) -1+x, y, z; (iii) -1+x, y, 1+z; (iv) 1-x, -y, 1-z; (v) 2-x, 1-y, z.]
